# Cpne7 deficiency induces cellular senescence and premature aging of dental pulp

**DOI:** 10.1111/acel.14061

**Published:** 2023-12-17

**Authors:** Yoon Seon Lee, Yeoung‐Hyun Park, Geumbit Hwang, Hyejin Seo, Si Hyoung Ki, Shengfeng Bai, Chul Son, Seong Min Roh, Su‐Jin Park, Dong‐Seol Lee, Ji‐Hyun Lee, You‐Mi Seo, Won Jun Shon, Daehyun Jeon, Mi Jang, Sahng G. Kim, Byoung‐Moo Seo, Gene Lee, Joo‐Cheol Park

**Affiliations:** ^1^ Laboratory for the Study of Regenerative Dental Medicine, Department of Oral Histology‐Developmental Biology School of Dentistry and Dental Research Institute, BK 21, Seoul National University Seoul Korea; ^2^ Department of Conservative Dentistry, School of Dentistry and Dental Research Institute Seoul National University Seoul Korea; ^3^ Regenerative Dental Medicine R&D Center HysensBio Co., Ltd. Gwacheon GyeonggiDo Korea; ^4^ Laboratory of Molecular Genetics, School of Dentistry and Dental Research Institute, BK 21 Seoul National University Seoul Korea; ^5^ Division of Endodontics Columbia University College of Dental Medicine New York New York USA; ^6^ Department of Oral and Maxillofacial Surgery, School of Dentistry and Dental Research Institute Seoul National University Seoul Korea

**Keywords:** aging, Copine7, dental pulp, oxidative stress, senescence, tooth

## Abstract

Once tooth development is complete, odontoblasts and their progenitor cells in the dental pulp play a major role in protecting tooth vitality from external stresses. Hence, understanding the homeostasis of the mature pulp populations is just as crucial as understanding that of the young, developing ones for managing age‐related dentinal damage. Here, it is shown that loss of *Cpne7* accelerates cellular senescence in odontoblasts due to oxidative stress and DNA damage accumulation. Thus, in *Cpne7*‐null dental pulp, odontoblast survival is impaired, and aberrant dentin is extensively formed. Intraperitoneal or topical application of CPNE7‐derived functional peptide, however, alleviates the DNA damage accumulation and rescues the pathologic dentin phenotype. Notably, a healthy dentin‐pulp complex lined with metabolically active odontoblasts is observed in 23‐month‐old *Cpne7*‐overexpressing transgenic mice. Furthermore, physiologic dentin was regenerated in artificial dentinal defects of *Cpne7*‐overexpressing transgenic mice. Taken together, Cpne7 is indispensable for the maintenance and homeostasis of odontoblasts, while promoting odontoblastic differentiation of the progenitor cells. This research thereby introduces its potential in oral disease‐targeted applications, especially age‐related dental diseases involving dentinal loss.

## Graphical abstract

Loss of Cpne7 leads to accelerated cellular senescence caused by oxidative stress and DNA damage accumulation in pulp cells, including odontoblasts. Treatment of CPNE7‐derived functional peptide (CPNE7‐DP) or Cpne7 transgenic mice rescues the aged dental pulp phenotype.

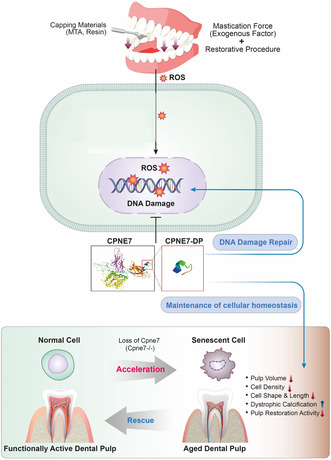

**Abbreviations**
ADSCs: adipogenic stem cells
BMSCs: bone marrow steml cells
BrdU: Bromodeoxyuridine
BSP: bone sialoprotein
CM: conditioned medium
Cpne7: Copine7
DCFDA: dichlorodihydrofluorescein diacetate
DP(S)Cs: dental pulp (stem) cells
DP: dervied peptide
DSP: dentin sialoprotein
GFs: gingival fibroblasts
HA/TCP: hydroxyapatite/tricalcium phosphate
KO: knock‐out
PCL: poly ε‐caprolactone
PDLSCs: periodontal ligament stem cells
ROS: reative oxygen species
SA‐β gal: Senescence‐associated β‐galactosidase
TG: transgenic
TUNEL: terminal deoxynucleotidyl transferase dUTP nick end labeling
WT: wild‐type

## 1 INTRODUCTION

As the globe meets the challenges of an aging population, there has been a growing interest in therapeutics for tooth injuries from various etiologies, such as dental caries and tooth abrasion. Tooth repair or regeneration requires a series of biological responses driven by multipotent cells comprising the dental pulp (Sloan & Smith, 2007). Odontoblasts are long‐lived post‐mitotic cells originating from neural‐crest‐derived ectomesenchyme and responsible for dentin development and eventually line dentin, a calcified tissue comprising nearly 70% of a healthy tooth that encloses the entire dental pulp (Nanci, 2017). Noticeably, odontoblasts elongate their cellular processes as they secrete and form predentin. Odontoblastic processes ultimately get embedded in the mineralized dentin matrix, establishing the dentinal tubule structure. After actively secreting the primary dentin until tooth eruption, these cells enter a metabolically less dynamic state where their secretory activities, the number of organelles, and the size of the cells all diminish (Couve et al., 2013). Mature, resting‐phase odontoblasts produce a minimal amount of dentin called secondary dentin throughout life. Sometimes, irritations such as carious lesions and dental restorative procedures stimulate the underlying resting odontoblasts to locally secrete tertiary dentin called reactionary dentin. If the irritations become too severe for existing odontoblasts to survive, cells in the sub‐odontoblastic layer differentiate into new odontoblasts and focally form the other type of tertiary dentin called reparative dentin (Smith et al., 2003). The characteristic life cycle of the odontoblast and its staged response to external stimuli contribute to protecting the pulp tissue. Thus, conserving the functions of odontoblasts is essential for keeping teeth protected and vital.

However, little is known about how odontoblasts age and survive through the days beyond entering dormancy, which actually comprise the major part of their life span. Aging is defined as a gradual loss of physiologic tissue integrity and impaired function, ultimately leading to cell death. Aged pulp is characterized by lower cell population, pulp stem cell tiredness, and, overall, reduced responsiveness to external stimuli (Bernick & Nedelman, 1975). It has been reported that one of the survival mechanisms of aging odontoblasts is autophagy, which involves degradation and turnover of cellular organelles (Couve, 1986). In our previous studies, we showed that *Cpne7*‐mediated autophagy modulates odontoblast process elongation, which may induce odontoblast‐like differentiation (Park et al., 2021). During tooth development, epithelial induction is necessary for the terminal differentiation of odontoblast progenitor cells. It was shown that Cpne7 is upregulated by TGFβ/BMP signaling in dental epithelium, then translocates to dental mesenchyme to promote odontoblast differentiation and dentin formation (Choung et al., 2016; Lee et al., 2011; Lee, Park, et al., 2022). Indeed, treating recombinant CPNE7 and its derivative peptide onto dentinal damage sites resulted in tertiary dentin formation of tubular nature (Lee et al., 2020).

In this study, we generated *Cpne7* knockout (*Cpne7* KO, *Cpne7* ^−/−^) mice to investigate the effects of *Cpne7* loss in the dentin and pulp environment. From 6 months of age, wild type (WT) mice started to form tubular tertiary dentin in response to natural tooth wear and aging. In contrast, *Cpne7* ^−/−^ mice displayed abnormal tertiary dentin with entrapped cells lacking the typical odontoblastic morphology. *Cpne7* ^−/−^ mice also demonstrated increased senescence and apoptosis of pulpal cells including odontoblasts, which was caused by highly elevated oxidate stress levels and accumulated DNA damage in the pulp. We further demonstrated that Cpne7 promotes DNA damage repair in the dental pulp cells. Lastly, CPNE7‐derived peptide injection was shown to rescue the pathologic phenotype of *Cpne7* ^−/−^ mice dentin‐pulp complex, while *Cpne7*‐overexpressing transgenic (*Cpne7* TG) mice were generated to examine the potential for the clinical application of Cpne7 in dentinal defects. Based on the current findings, we suggest that Cpne7 plays an essential role in maintaining the functions of mature odontoblasts after inducing odontoblast differentiation.

## 2 RESULTS

### 2.1 Cpne7 promotes odontoblastic differentiation of progenitor cells in the dental pulp

**FIGURE 1:**
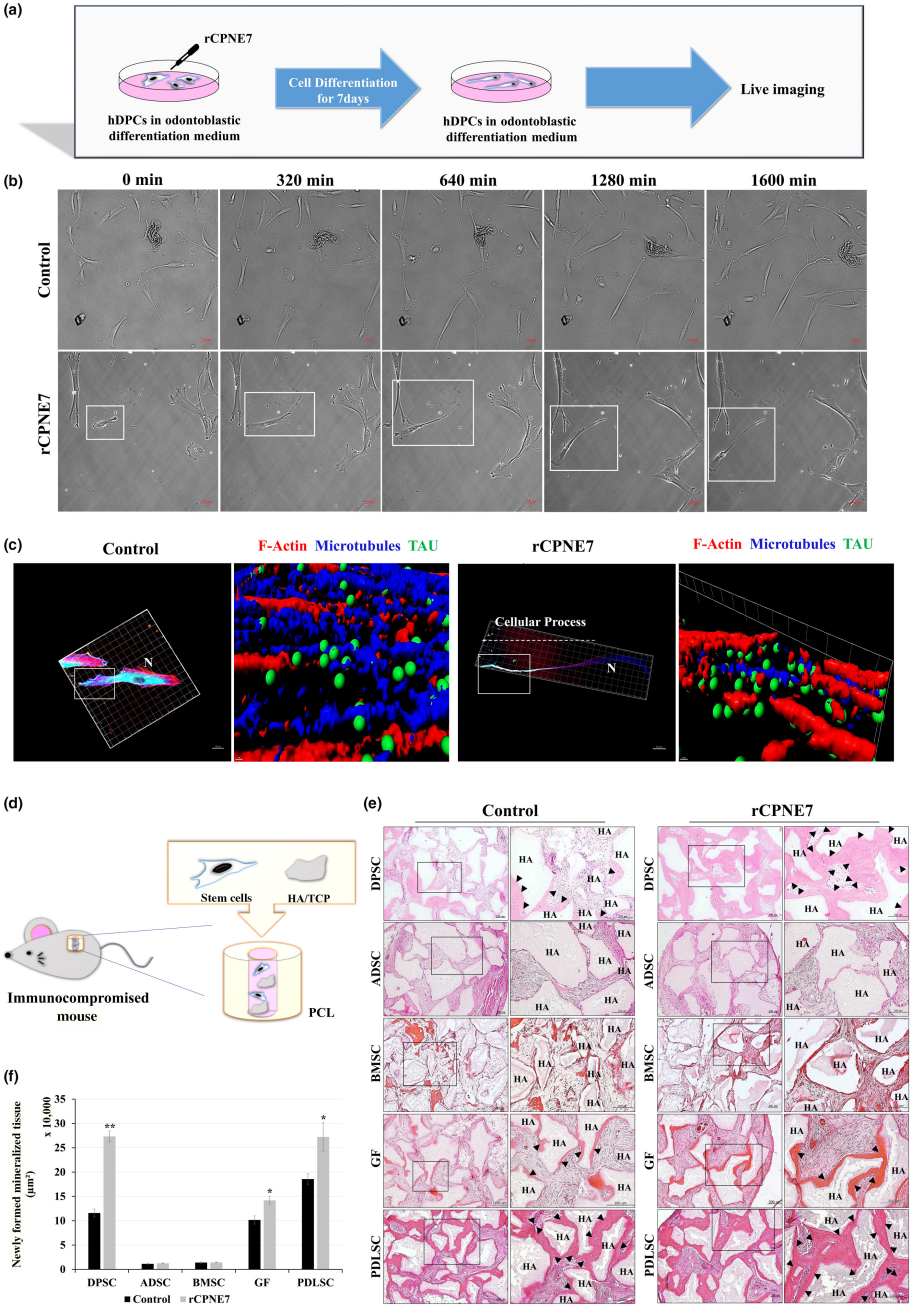
Cpne7 Promotes odontoblastic differentiation of progenitor cells in the dental pulp. (a) Schematic diagram of the live‐imaging in recombinant CPNE7 (rCPNE7)‐treated hDPCs. (b) Live‐cell images of hDPCs cultured in differentiation media for 7 days following 24 h of rCPNE7 treatment. Each frame was collected from a single Z plane, and recorded at 320 min intervals. Odontoblast process‐like structure elongation (white boxed area) is observed in rCPNE7‐treated hDPCs. Scale bars: 50 μm. (c) Snapshots at various points on the Z plane, which were generated in three dimensions using the Imaris software. Localizations of TAU (green sphere), F‐Actin (red), and Microtubule (blue) in hDPCs are shown. Boxed area on the left is shown at a higher magnification on the right. N, Nucleus. (d) Schematic diagram of ex vivo subcutaneous transplantation model. Stem cells were mixed with 100 mg of hydroxyapatite/tricalcium phosphate (HA/TCP) particles alone (control) or with rCPNE7 in a poly ε‐caprolactone (PCL) spaces and transplanted subcutaneously into immunocompromised mice for 6 weeks. (e) H&E staining of the sectioned samples was analyzed histologically. Boxed areas on the left (scale bars: 200 μm) Are shown at higher magnification on the right (scale bars: 200 μm). Black arrowheads indicate newly formed mineralized tissues. The dental pulp stem cells (DPSCs), adipogenic stem cells (ADSCs), bone marrow stem cells (BMSCs), gingival fibroblasts (GFs), and periodontal ligament stem cells (PDLSCs) were mixed with 100 mg of HA/TCP particles alone (control) or with rCPNE7 in a poly ε‐caprolactone (PCL) spaces and transplanted subcutaneously into immunocompromised mice for 6 weeks. (f) Semi‐quantification of the volumes of the newly mineralized tissues. All values represented the mean ± SD of triplicate experiments. **p < 0.01 and *p < 0.05 vs. Control.

We previously reported that recombinant CPNE7 (rCPNE7) treatment promoted odontoblast differentiation and physiologic dentin formation retaining the tubular structure (Choung et al., 2016). Live‐cell imaging of human dental pulp cells (hDPCs) with or without rCPNE7 treatment was taken at an 8‐min interval (Video S1), and images were exported at a 320‐min interval. Unlike in the control group, where cells extended in an irregular manner, elongation of a uni‐directional cellular process was observed in the rCPNE7‐treated group (Figure 1a,b). Furthermore, the expression of microtubule‐associated protein Tau (MAPT), a newly suggested morphological marker of odontoblast (Miyazaki et al., 2015), co‐localized with the linker regions of F‐Actin and α‐Tubulin onto the labile end of the elongating cellular process both in the rCPNE7‐treated and control group (Figure 1c). Consistently, rCPNE7‐treated hDPCs showed enhanced odontoblast‐like differentiation and dentin‐like tissue‐forming capacity, compared to the control group ex vivo (Figure 1d–f). As Cpne7 has been shown to promote odontoblastic differentiation of hDPCs followed by dentin‐like tissue formation, we next examined its effects in non‐odontogenic stem cells, such as adipogenic stem cells (ADSCs), bone marrow stem cells (BMSCs), gingival fibroblasts (GFs), and periodontal ligament stem cells (PDLSCs). Noticeably, rCPNE7‐treated GFs and PDLSCs showed a slightly greater amount of newly formed DSP‐positive mineralized tissue than the control groups in poly ε‐caprolactone (PCL) spaces (Figure 1e,f and Figure S1). These results suggest that cells of different lineages display different responses to Cpne7, and the odontoblastic induction potential of Cpne7 is specifically effective in the odontoblast progenitor cells of the dental pulp environment.

### 2.2 Pathologic tertiary dentin is deposited in *Cpne7*‐null mouse molar

Next, in order to evaluate the consequences of loss of function, *Cpne7*‐null mice were generated. The targeting construct included an *EGFP* coding region and Neomycin‐resistance cassette (*NeoR*), in place of the coding sequence for *Cpne7* from exons 5 to 7 (Figure S2a). The construct was electroporated into the CMTI‐1 ES cell line derived from mouse strain 129/SVEV. After electroporation of the construct into ES cell line, G418/ganciclovir‐resistant colonies were screened by PCR using the primers covering both the insert and the backbone (Figure S2b). The targeted cells yielded 3.45‐kb, 2.67‐kb, and 2.40‐kb PCR products, according to the primer sets designed. Dental pulp cells of the WT and *Cpne7* ^−/−^ mice were immunohistochemically analyzed to confirm the loss of CPNE7 protein expression at postnatal 1 month (Figure S2c). Body weight measurement showed no significant difference until 12 weeks, between WT and *Cpne7* ^−/−^ mice (Figure S2d).

**FIGURE 2:**
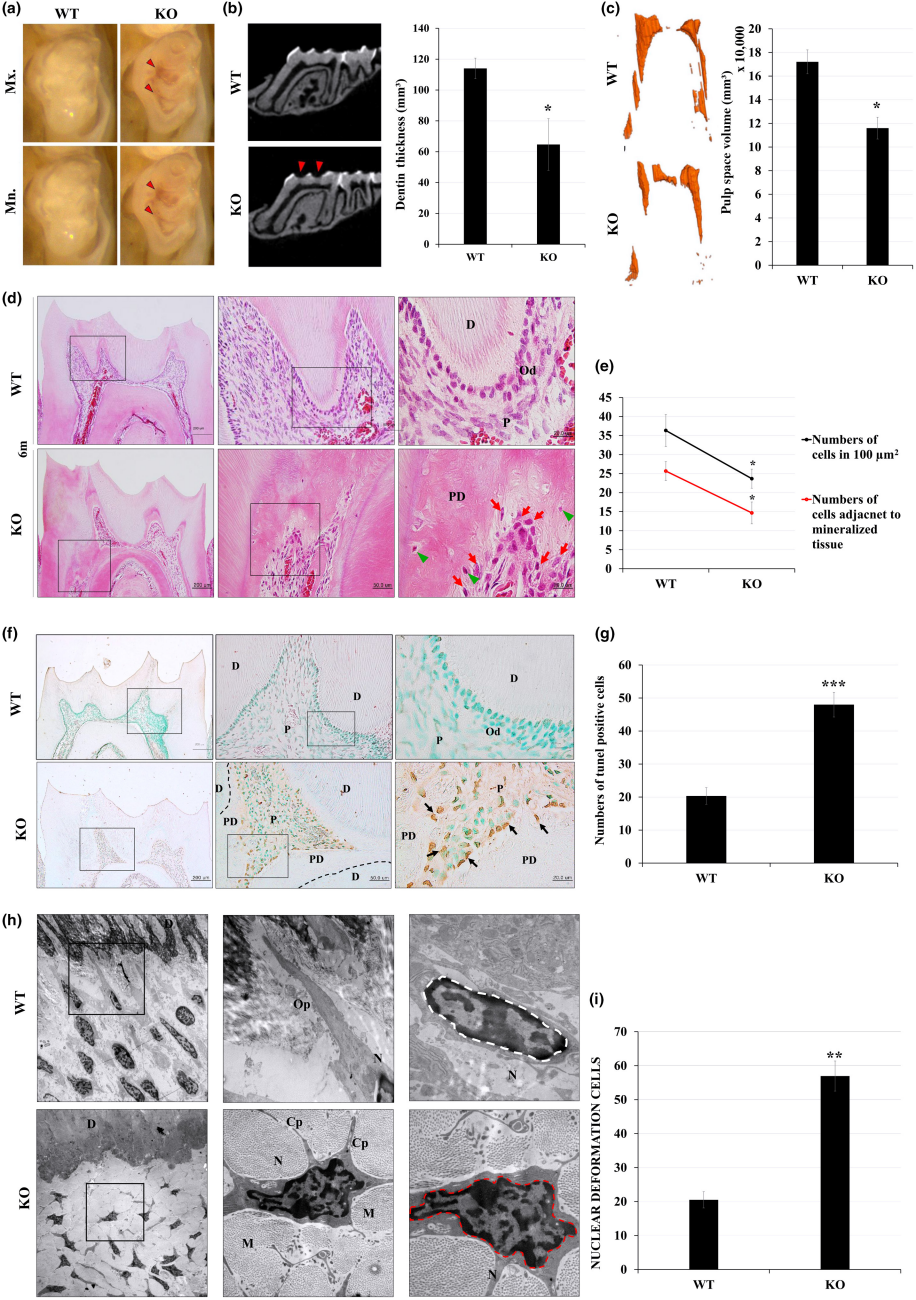
*Cpne7*‐null mice molars demonstrate pathologic dentin‐pulp complex phenotype. (a) Light microscopic images of 6‐month‐old WT and *Cpne7* ^−/−^ mice molars. Thinner coronal dentin (red arrowheads) through which the underlying pulp chambers are seen in *Cpne7* ^−/−^ mice. Mx, Maxilla; Mn, Mandible. (b) Micro‐computed tomographic images of WT and *Cpne7* ^−/−^ mice mandibles manifest thinner coronal dentin (red arrowheads) in *Cpne7* ^−/−^ mice. Quantitative analysis of coronal dentin thickness in WT and *Cpne7* ^−/−^ mice molars. (c) 3D reconstruction of pulp spaces and quantitative analysis of pulp space volumes in WT and *Cpne7* ^−/−^ mice molars. (d) Histological analysis of mandibular first molars of WT and *Cpne7* ^−/−^ mice at 6 months by H&E staining. Scale bars: 200 μm. Boxed areas are shown at higher magnification. Scale bars: 50 μm, 20 μm. D, Dentin; Od, Odontoblasts; P, Dental pulp; PD, Pathologic dentin; green arrowhead, entrapped cells; red arrow, cells getting entrapped. (e) Cell counting analysis at the dentin‐pulp interfaces of WT and *Cpne7* ^−/−^ mice molars. (f) TUNEL staining of dental pulp cells of WT and *Cpne7* ^−/−^ mice at 6 months. Scale bars: 200 μm. Boxed areas are shown at higher magnification. Scale bars: 50 μm, 20 μm. D, Dentin; Od, Odontoblasts; P, Dental pulp; PD, pathologic dentin; black arrow, TUNEL‐positive cells. (g) Quantitative analysis of the number of TUNEL‐positive cells in WT and *Cpne7* ^−/−^ mice molars. (h) Transmission electron microscopic images of dentin‐pulp interfaces in the WT and *Cpne7* ^−/−^ mice molars. Normal nuclear‐shaped hDPCs (white dotted line) and nuclear‐deformed hDPCs (red dotted line) are observed in WT and *Cpne7* ^−/−^ mice molars, respectively. D, dentin; Op, odontoblast process; N, nucleus; M, mineralized tissue; Cp, cytoplasmic process. (i) Semi‐quantification of nuclear deformation in WT and *Cpne7* ^−/−^ odontoblasts. All values represented the mean ± SD of triplicate experiments. **p < 0.01 and *p < 0.05 vs. Control.

Maxillary and mandibular first molars of *Cpne7* ^−/−^ mice at 6 months manifested thinner coronal dentin through which the underlying pulp chambers were seen (Figure 2a). Micro‐CT images also showed that dentin thickness was thinner in *Cpne7* ^−/−^ mice compared to WT (Figure 2b). Also, a narrower pulp volume was observed in *Cpne7* ^−/−^ mice compared to WT (Figure 2c). Histological analysis was performed to investigate changes in the dentin‐pulp complex. Normal tooth development was observed in tooth germs of *Cpne7* ^−/−^ mice at late bell stage of embryonic 18 days (E18) (Figure S3). Primary and secondary dentin of *Cpne7* ^−/−^ mice seemed to develop normally like those of WT (Data not shown). At 6 months, however, the *Cpne7* ^−/−^ mice had a disruption of odontoblast alignment and an evident decrease in pulp cell population (Figure 2d,e). In WT, columnar‐shaped odontoblasts regularly lined the dentin, whereas, in *Cpne7* ^−/−^ mice, more cuboidal‐shaped odontoblasts were non‐uniformly arranged in the dental pulp (Figure 2d). Moreover, the radicular pulp of *Cpne7* ^−/−^ mice was partly obliterated with abnormal bone‐like tissue. Noticeably, round‐shaped cells were entrapped in the lacunae‐like spaces in this pathologic dentin (PD) structure. We next checked the expression of dentin sialoprotein (DSP) and bone sialoprotein (BSP), the respective dentin and bone marker proteins by immunohistochemical staining of the dentin‐pulp complex. DSP showed much weaker staining for odontoblasts in *Cpne7* ^−/−^ than in WT and the embedded cells stained specifically positive for BSP, a major constituent of bone matrix (Figure S4). Also, a narrow dental pulp volume and reduced dental pulp cell population were observed in *Cpne7* ^−/−^ mice compared to the WT (Figure 2e,f). A similar pathologic condition was also observed in the maxillary molars of 6‐month‐old *Cpne7* ^−/−^ mice (Figure S5). These results suggest that in the absence of *Cpne7*, pathologic tertiary dentin showing bone‐like characteristics is formed in the dental pulp.

### 2.3 Cell survival is impaired in *Cpne7*‐null mouse molar

To investigate whether the number of cells comprising the *Cpne7* ^−/−^ pulp decreased due to cellular apoptosis, we performed TUNEL assay (Figure 2f). While most odontoblasts in the WT mouse pulp were TUNEL‐negative, positive signals were diffusely located in the odontoblast layer of the *Cpne7* ^−/−^ mouse pulp (Figure 2g). Moreover, the cells embedded in the dentin matrix, which had lost their morphological polarities, were also TUNEL‐positive. Whether these cells are dead odontoblasts that have been incorporated into the tertiary dentin or newly differentiated odontoblast‐like cells was unclear, so their origins were traced by BrdU labeling. An analog of the nucleoside thymidine, BrdU is integrated into replicating DNA and can be used to identify proliferating cells. The intraperitoneal injection of BrdU was made at 4 months, and staining pattern was analyzed at 6 months. Since odontoblasts are non‐dividing post‐mitotic cells, we expected that BrdU would only label those originating from actively dividing pulp cells. Anti‐BrdU antibodies were detected in the entrapped round‐shaped cells in *Cpne7* ^−/−^ mouse tertiary dentin as well as in few of the pulp cells in both WT and *Cpne7* ^−/−^ mice (Figure S6). We next examined the morphologies of odontoblast‐like cells underlying newly formed pathologic dentin through transmission electron microscopy. Typical odontoblast processes with regular elongation from the cell body were observed in the WT, which were inserted into the dentinal tubule structure. However, the morphology of multiple cellular extensions stemming from a single cell body was observed in *Cpne7* ^−/−^ (Figure 2h). Moreover, most of these abnormal cells were entrapped in the mineralized tissue. Another notable feature was a greater number of cells with nuclear deformation in *Cpne7* ^−/−^ than in WT (Figure 2i). These results suggest that odontoblasts in *Cpne7* ^−/−^ mice undergo apoptosis at some point, and in response to external stimuli like attrition, new pulp cells are induced to differentiate toward odontoblast‐like cells deprived of cellular properties of odontoblasts to form pathologic tertiary dentin, and eventually die after their entrapments.

### 2.4 *Cpne7* deletion leads to premature aging of mouse molar pulp environment

**FIGURE 3:**
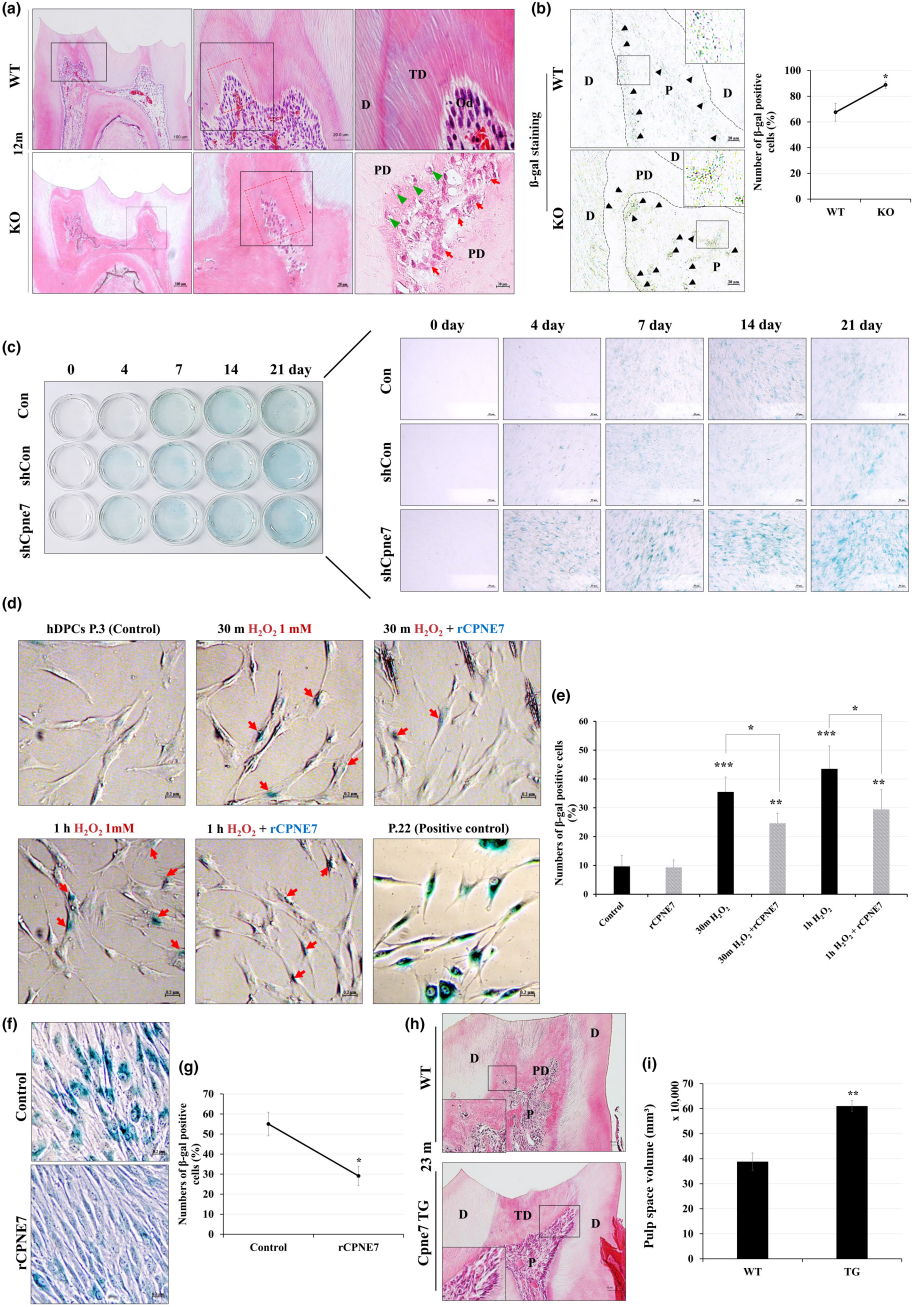
*Cpne7* deletion leads to premature aging of the mouse molar pulp environment. (a) Histological analysis of mandibular first molars of WT and *Cpne7* ^−/−^ mice at 1 year by H&E staining. Scale bars: 200 μm. Boxed areas are shown at higher magnification. Scale bars: 50 μm, 20 μm. D, Dentin; Od, Odontoblasts; P, Dental pulp; PD, Pathologic dentin; green arrowhead, entrapped cells; red arrow, cells getting entrapped. (b) Senescence‐associated β‐galactosidase (SA‐ β gal) activity (black arrowhead) is observed in 6‐month‐old WT and *Cpne7* ^−/−^ mice molar by light microscope. Quantitative analysis of the number of SA‐β gal positive cells in WT and *Cpne7* ^−/−^ mice molars. Scale bars: 20 μm. Boxed areas are shown at higher magnification. D, Dentin; P, Dental pulp; PD, Pathologic dentin. (c) Human DPCs were transfected with shControl (shCon) or shCpne7 vectors for 48 h and cultured in differentiation medium for up to 21 days. Representative images of SA‐β gal activity in Control, shCon, or shCpne7 groups. (d) The hDPCs (passage 3) were treated with 1 mM H_2_O_2_ or H_2_O_2_ + rCPNE7 for 30 min or 1 h. The hDPCs (passage 22) were used as positive control. SA‐β gal activity (red arrowhead) is observed in H_2_O_2_ only or with rCPNE7‐treated hDPCs. (e) Semi‐quantification of SA‐β gal activity was analyzed. (f) The hDPCs (passage 3) were treated with 1 mM H_2_O_2_ or H_2_O_2_ + rCPNE7 for 72 h. SA‐β gal activity is observed in H_2_O_2_ only or with rCPNE7‐treated hDPCs. (g) Quantitative analysis of the number of SA‐β gal positive cells. (h) Histological analysis of mandibular first molars of WT and *Cpne7* TG mice at 23 months by H&E staining. Scale bars: 100 μm. Boxed areas are shown at higher magnification. Scale bars: 50 μm. D, Dentin; TD, Tertiary dentin; P, Dental pulp; PD, Pathologic dentin. (i) Quantitative analysis of the pulp space volumes in WT and *Cpne7* TG mice molars at 23 months. All values represented the mean ± SD of triplicate experiments. *p < 0.05, **p < 0.01, and ***p < 0.001 vs. Control.

The phenotypic changes became more apparent in 12‐month‐old *Cpne7* ^−/−^ mice. Extensive obliteration of the pulp space due to accelerated tertiary dentin deposition was exhibited (Figure 3a). While the typical palisade layer of odontoblasts was preserved in the WT, it completely collapsed in *Cpne7* ^−/−^ mouse. Further, the pulp cells became even more sparsely distributed, some of which being entrapped within the newly‐formed pathologic dentin. Since cellular senescence is closely related to age‐related abnormalities, we next checked whether loss of *Cpne7* triggered cellular senescence in the pulp. As shown in Figure 3b, more intense senescence‐associated beta‐galactosidase (SA‐β gal) activity was observed in *Cpne7* ^−/−^ pulp. Cellular senescence level during the odontoblastic differentiation of hDPCs with or without knocking down *Cpne7* was investigated in vitro. *Cpne7* mRNA expression was most effectively suppressed after transfecting the shCpne7 vector at the concentration of 7000 ng/mL (Figure S7a,b). Following shCpne7 transfection, hDPCs were cultured in the odontogenic differentiation medium for up to 21 days, and SA‐β gal activity was measured in each group. More senescent cells were detected toward the late stage of differentiation in all groups, but a remarkably greater number of SA‐β gal‐positive cells were observed in the *Cpne7* knock‐down group (Figure 3c).

Next, we evaluated the role of exogenous Cpne7 in repressing cellular senescence in dental pulp cells. Oxidative stress is a commonly utilized driver of cellular senescence, so we used hydrogen peroxide (H_2_O_2_) to induce cellular senescence in vitro. SA‐β gal positive signals began to deposit in hDPCs 30 min after 1 mM H_2_O_2_ treatment (Figure 3d,e). Unlike the H_2_O_2_‐only treated group, the number of SA‐β gal‐positive dental pulp cells was significantly decreased after treating rCPNE7 an hour later. Human DPCs sub‐cultured to the 22nd passage was used as a positive control for senescence. In hDPCs cultured in growth media containing rCPNE7 for 72 h, cellular senescence progressed more slowly than the control after exerting oxidative stress for an hour (Figure 3f,g).

Furthermore, *Cpne7* over‐expressing TG mice were generated using *keratin‐14* (*K14*) promoter (Figure S8a), and raised for 2 years. Considering that endothelial cells also express *K14*, we expected CPNE7 secreted from the blood vessels of *K14‐Cpne7* TG mice could circulate to affect the dental pulp. The expression level of CPNE7 was significantly higher in the blood drawn from *K14‐Cpne7* TG mice than in WT, according to ELISA analysis (Figure S8b). In the absence of *Cpne7*, wild‐type mice at 23 months showed a similar dental pulp phenotype to that of 6‐month‐old *Cpne7* ^−/−^ mice, confirming the premature aging of the dentin‐pulp complex (Figure 3h). Constriction of the pulp space by pathologic dentin accumulation, and loss of odontoblast alignment were evident. Interestingly, 23‐month‐old *Cpne7* TG mice demonstrated a larger pulp chamber area with the typical palisade layer of odontoblast lining the dentin (Figure 3i). Partial obliteration of radicular pulp and formation of physiologic tertiary dentin in response to attrition were also observed.

Morphological changes of incisor in *Cpne7* ^−/−^ mice were analyzed by both light and electron microscopies. However, histologically, no noticeable changes were detected in mandibular incisors of 6‐ and 12‐month‐old mice (Figure S9a,b). Micro‐CT images also showed no obvious differences between *Cpne7* ^−/−^ and WT for incisors (Figure S9c). Next, we identified the genetic level differences in dental pulp cells including odontoblasts isolated from mandibular incisors. RNA sequencing data confirmed that *ApoE* and *Klhdc4* genes were significantly upregulated, while *Ces1g*, *Fgf10*, and *Mapt* genes were downregulated (Figure S9d). Certain genes involved in odontogenesis, *Dmp1*, *Mapt*, and *Wnt6* were also downregulated when *Cpne7* was absent (Figure S9d). Mouse incisor has the distinct characteristic of permanent growth throughout life by continuous interaction between dental epithelial and mesenchymal cells. Such a difference between mouse incisor and molar suggests the possibility that other factors in dental epithelium could compensate for the loss of function of Cpne7. To confirm the hypothesis, we isolated apical bud cells (ABCs) from incisors of WT and KO mice and obtained conditioned‐medium (CM) (Figure S9e,f). After each CM was treated to dental pulp cells, the expression levels of odontoblast differentiation marker proteins like DSP, DMP‐1, RUNX‐2, and NESTIN were identified by western blotting. Interestingly, DSP and DMP‐1 increased in WT CM and KO CM‐treated groups compared to the non‐treated group (Control), but there was no significant difference between each CM‐treated group (Figure S9g,e). These results suggest that other dental epithelial factors that could compensate for the loss of function of Cpne7 are present in the mouse incisor environment.

### 2.5 Cpne7 modulates intracellular ROS level in dental pulp cells

**FIGURE 4:**
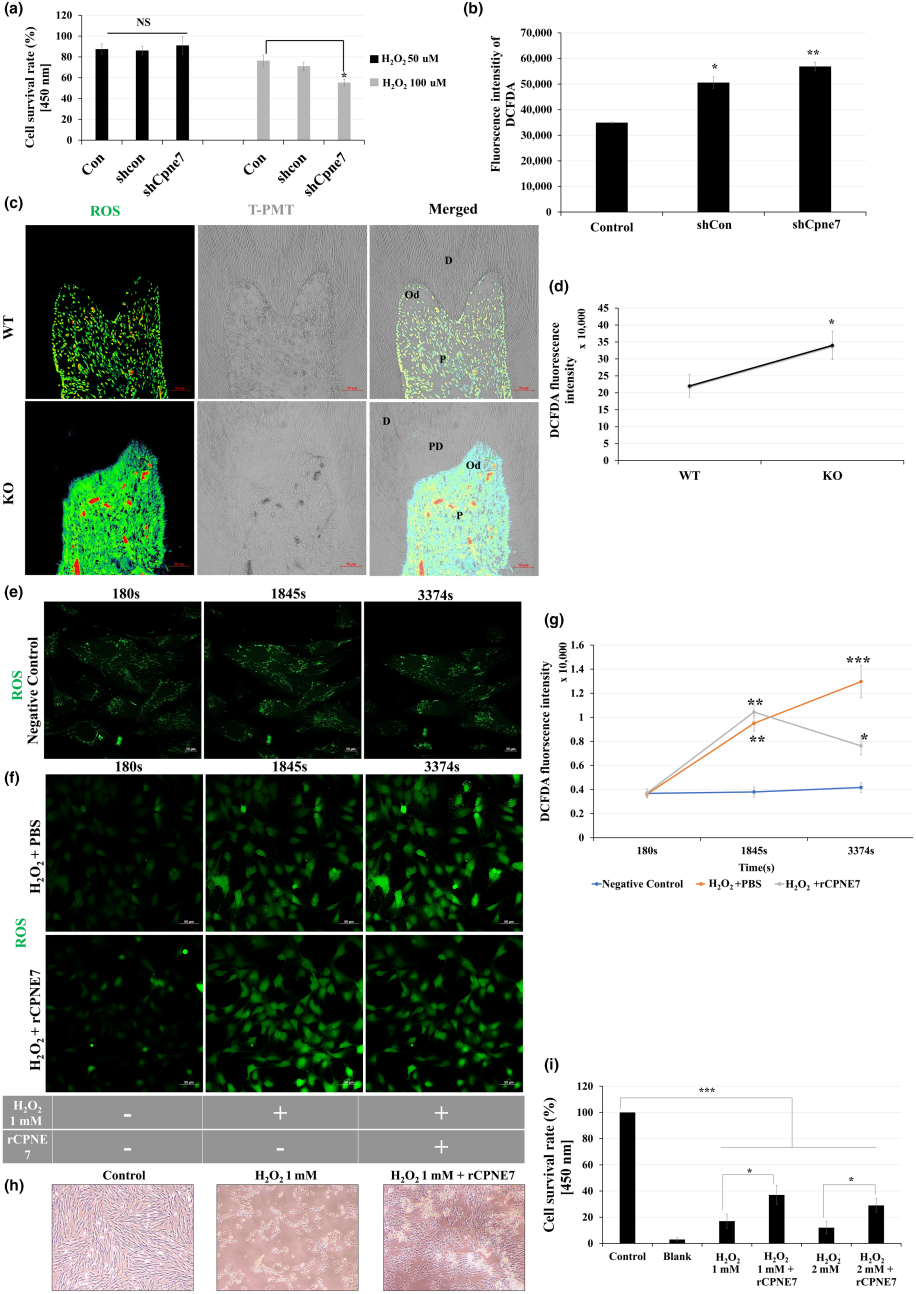
Cpne7 modulates intracellular reactive oxygen species (ROS) level in dental pulp cells. (a) Human DPCs were transfected with shCon or shCpne7; 48 h later, hDPCs were treated with 50 μM or 100 μM H_2_O_2_ for 24 h and WST‐8 cell viability assay was performed. Oxidative stress‐induced cytotoxicity was analyzed by WST‐8 cell viability assay in hDPCs. (b) Control, shCon, or shCpne7 group incubated with DCFDA for 1 h and microplate analysis of green fluorescent DCFDA signals. (c) DCFDA signal was analyzed in 6‐month‐old WT and *Cpne7* ^−/−^ mice molar by confocal microscopy. Scale bars: 50 μm. D, Dentin; Od, Odontoblasts; P, Dental pulp; PD, Pathologic dentin (d) Semi‐quantification of DCFDA signal was analyzed in 6‐month‐old WT and *Cpne7* ^−/−^ mice molar. (e) Live cell imaging of base intracellular ROS level in hDPCs through DCFDA staining. (f) Live cell imaging of intracellular ROS level by rCPNE7 treatment 15 min after H_2_O_2_ treatment in hDPCs through DCFDA staining. Messenger RNA level of *Cpne7* was analyzed by RT‐PCR at 30 min or 1 h after H_2_O_2_ 100 μM, 500 μM, or 1 mM treatment in hDPCs, respectively. (g) DCFDA fluorescence intensity was analyzed. (h) Light microscopic images of hDPCs treated with H_2_O_2_ only or with rCPNE7. (i) Cytotoxicity was analyzed by WST‐8 cell viability assay in hDPCs treated with H_2_O_2_ only or with rCPNE7. All values represented the mean ± SD of triplicate experiments. *p < 0.05 and **p < 0.01 vs. Control.

Excessive reactive oxygen species (ROS) and free radicals such as superoxide, H_2_O_2_ generate oxidative stress that can eventually cause cellular damage like senescence and apoptosis (Harraan, 1955). When hDPCs were treated with H_2_O_2_ for 24 h, cell death occurred in a concentration‐dependent manner except for 50 μM (Figure S10a). There was also no difference in cell survival rate in H_2_O_2_ 50 μM‐treated hDPCs in the ShCpne7 group compared to the control groups (Figure 4a). However, the cell survival rate was significantly reduced in the shCpne7 group at concentrations above 100 μM of H_2_O_2_ (Figure 4a). On the other hand, the basal level of ROS was relatively higher than that of the control groups (Figure 4b). In addition, higher ROS level was observed in the dental pulp environment in 6‐month‐old KO mice molar than in WT in vivo (Figure 4c,d). Interestingly, the endogenous mRNA expression level of *Cpne7* started to increase after treatment of H_2_O_2_ in hDPCs (Figure S10b,c). We next identified whether Cpne7 is involved in the modulation of artificially induced excessive intracellular ROS levels. Live‐cell imaging of hDPCs with or without treating rCPNE7 after H_2_O_2_ treatment was taken at a 1‐min interval (Video S2), and images were exported at a 60‐min interval. In the 1× PBS‐treated group after treatment of H_2_O_2_, the intracellular ROS level gradually increased in a time‐dependent manner compared to untreated hDPCs (Negative control) (Figure 4e,f, Video S2). Interestingly, however, the intracellular ROS level, which increased in a time‐dependent manner, gradually decreased back to the normal level (Figure 4f,g, Video S2). We next identified the effect of exogenous rCPNE7 treatment on ROS production. Treating 1 mM and 2 mM of H_2_O_2_‐induced oxidative stress resulted in severe cytotoxicity, and most of the cell‐to‐cell contact was disorganized after 48 h (Figure 4h,i). However, in the rCPNE7‐treated group after 30 min treatment of H_2_O_2,_ the cell viability was higher, and the cell‐to‐cell contact was more organized than that of the H_2_O_2_ only treated group (Figure 4h,i). These results suggest that Cpne7 may be responsible for the maintenance of intracellular ROS levels.

### 2.6 Deletion of *Cpne7* leads to DNA damage accumulation in dental pulp cells

**FIGURE 5:**
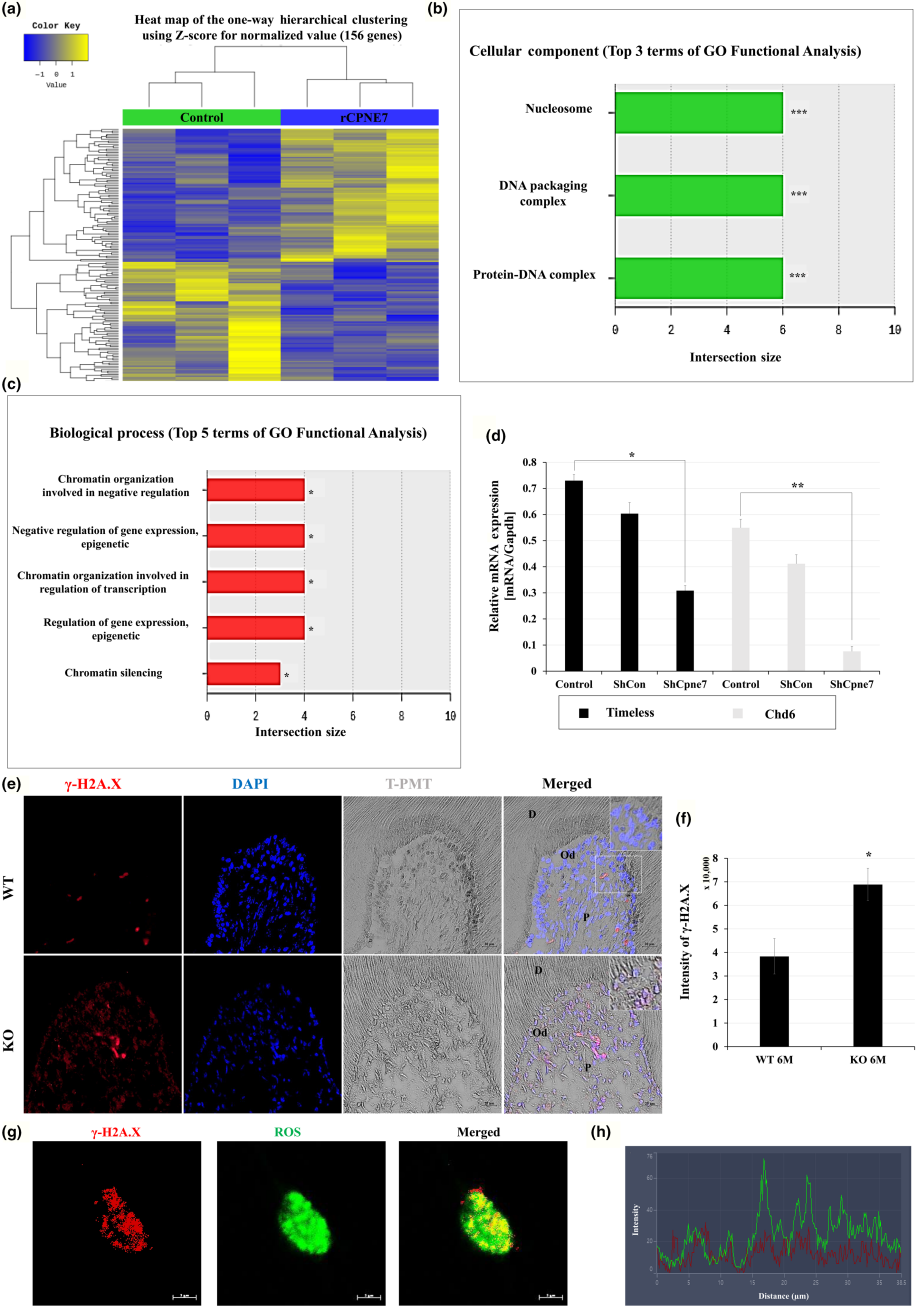
*Cpne7* deletion leads to DNA damage accumulation in dental pulp cells. (a–c) Heat map construction for hierarchical clustering and a list of downregulated or upregulated genes in rCPNE7‐treated hDPCs. (d) Human DPCs were transfected with shCon or shCpne7 for 48 h. *Timeless* and *Chd6* mRNA levels were evaluated by qPCR. (e) Representative immunofluorescence images of γ‐H2AX (red) and semi‐quantification in 6‐month‐old WT and *Cpne7* ^−/−^ mice molar (*n* = 3). DAPI (blue) was counterstained to indicate the nucleus. Scale bars: 20 μm. Boxed areas are shown at higher magnification. D, Dentin; Od, Odontoblasts; P, Dental pulp. (f) Semi‐quantification of γ‐H2AX (red) signal was analyzed. (g, h) Representative immunofluorescence images of γ‐H2AX (red) and ROS (green) in hDPCs (*n* = 3). DAPI (blue) was counterstained to indicate the nucleus. Semi‐quantification of γ‐H2AX (red) and ROS (green) signal was analyzed in hDPCs. All values represented the mean ± SD of triplicate experiments. *p < 0.05, **p < 0.01, and ***p < 0.001 vs. Control.

Next, we conducted RNA‐sequencing analysis to identify the detailed functions or cellular involvement of Cpne7 in hDPCs (Figure 5a). According to GO functional analysis, Cpne7 is closely related to the biological process of chromatin organization. In addition, Cpne7 was shown to have a close connection with cellular components like nucleosome, DNA packaging complex, and protein‐DNA complex (Figure 5b,c). Consistent with the functional analysis, the mRNA expression levels of various histone and chromatin‐related genes were significantly increased in rCPNE7‐treated hDPCs (Figure S11). Organizing a chromosome, histones mark the site of DNA damage to recruit a number of DNA damage repair proteins including Timeless and Chromodomain helicase DNA‐binding protein‐6 (*Chd6*) (Moore et al., 2019; Young et al., 2015). Indeed, the mRNA expression levels of *Timeless* and *Chd* *6* were significantly decreased in the ShCpne7 group (Figure 5d). Furthermore, the expression of γ‐H2AX, DNA damage marker protein (Pilch et al., 2003), was relatively more detected in dental pulp cells of *Cpne7* ^−/−^ mouse molar at 6‐month compared to WT in vivo (Figure 5e,f). We next investigated whether DNA damage is induced by oxidative stress in hDPCs. After generating oxidative stress by treating H_2_O_2_, immunofluorescence was performed with the γ‐H2AX in hDPCs. The expression of γ‐H2AX and ROS were co‐localized in the nucleus of hDPCs (Figure 5g,h). These results suggest that the increase in the ROS level of dental pulp cells caused by *Cpne7* deletion may be related to DNA damage accumulation.

### 2.7 Cpne7 is required for DNA damage response and repair process in dental pulp cells

**FIGURE 6:**
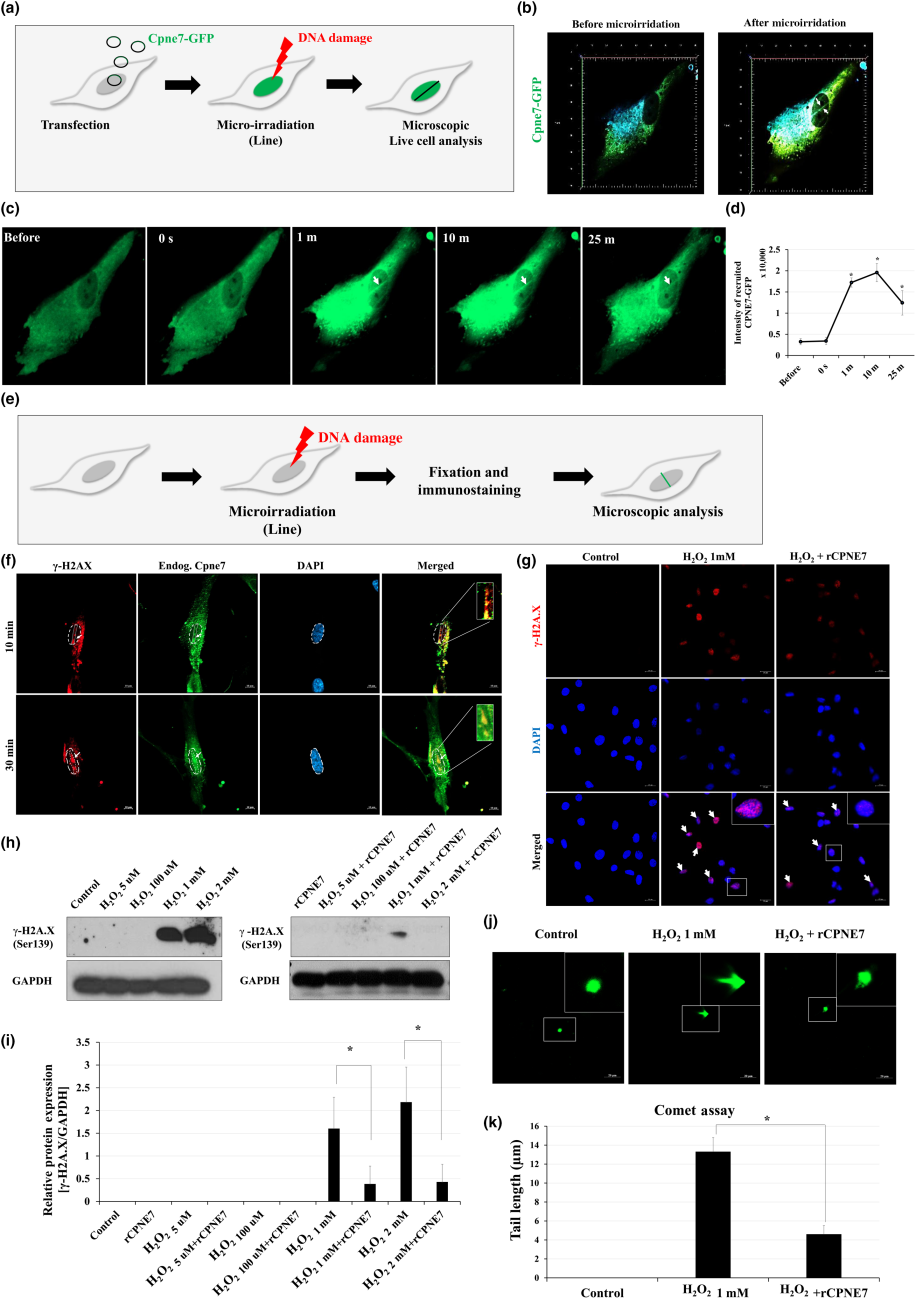
Cpne7 is involved in the repair process of oxidative stress‐induced DNA damage in dental pulp cells. (a) Schematic diagram of the microirradiation model in *Cpne7*‐GFP transfected hDPCs. (b) Expression level of CPNE7 and its localization (white arrowheads) before and after microirradiation in hDPCs. (c) Time course images of CPNE7 recruitment (white arrowheads) to the sites of DNA damage in hDPCs. (d) Semi‐quantification of GFP‐tagged CPNE7 signal at each time point. (e) Schematic diagram of the microirradiation model in hDPCs. (f) The recruitment of endogenous CPNE7 (green) was detected 10 and 30 min after DNA damage induction and co‐localized with γ‐H2AX (red). DAPI (blue) was counterstained to indicate the nucleus. Scale bars: 10 μm. Boxed areas are shown at higher magnification. (g) Representative immunofluorescence images of γ‐H2AX (white arrow) in H_2_O_2_ only or with rCPNE7‐treated hDPCs. DAPI (blue) was counterstained to indicate the nucleus. Scale bars: 20 μm. Boxed areas are shown at higher magnification. (h, i) The hDPCs were treated with 5 μM, 100 μM, 1 mM, or 2 mM of H_2_O_2_ only or w H_2_O_2_ + rCPNE7 for 24 h, respectively. Gamma‐H2AX protein levels were evaluated by western blot analysis and semi‐quantified. (j, k) Neutral comet assay of hDPCs, treated for 24 h with H_2_O_2_ only or H_2_O_2_ + rCPNE7. Comet images of the hDPCs (green) were observed under a confocal microscope and semi‐quantified. Scale bars: 20 μm. Boxed areas are shown at higher magnification. All values represented the mean ± SD of triplicate experiments. *p < 0.05 vs. Control.

Then, we examined whether DNA damage alters intracellular localization of CPNE7. Green fluorescent protein (GFP) tagged *Cpne7* (*Cpne*7^GFP^) construct was transfected into hDPCs, and microirradiation was induced specifically to the cellular nucleus by confocal microscopy (Figure 6a). To identify the spatio‐temporal distribution of CPNE7 at DNA damage sites in real time, we performed live‐cell imaging analysis of hDPCs expressing GFP‐tagged CPNE7 (Video S3). Interestingly, CPNE7^GFP^ started to get recruited to the laser‐induced DNA damage sites after 1 min and gradually faded out after 25 min, suggesting an early role of Cpne7 in the DNA damage response (DDR) (Figure 6b–d). Furthermore, endogenous CPNE7 was also detected along the DNA damage tracks, which overlapped with γ‐H2AX after 10 min of microirradiation (Figure 6e,f). The evidence of co‐localization became faint after 30 min of microirradiation (Figure 6f).

We next investigated the possibility of Cpne7 being involved in the DNA damage repair process. To evaluate the role of Cpne7 in DNA damage repair, we treated rCPNE7 to hDPCs after 30 min of H_2_O_2_ treatment. The expression of γ‐H2AX was specifically detected in cellular nuclei of hDPCs after treatment of 1 mM H_2_O_2,_ but not in the control group (Figure 6g). However, accumulation of DNA damage was rarely observed in the rCPNE7‐treated group after H_2_O_2_ treatment compared to H_2_O_2_‐only treated group (Figure 6g). Consistently, the protein expression level of γ‐H2AX was clearly detected at 1 mM and 2 mM H_2_O_2_‐treated groups in western blot analysis (Figure 6h,i). Interestingly, after 48 h, the protein expression level of γ‐H2AX was hardly detected in the rCPNE7‐treated groups compared to the H_2_O_2_‐only treated groups (Figure 6h,i). In addition, we could observe the fragmented DNA visualized as a cell tail, after treatment of 1 mM H_2_O_2_ through comet assay (Figure 6j,k). The length of the tail was also much shorter in the rCPNE7‐treated group, suggesting that Cpne7 may play a crucial role in the repair process for oxidative stress‐induced DNA damage.

### 2.8 CPNE7‐derived functional peptide rescues the pathologic phenotype of *Cpne7* ^−/−^ mice dentin‐pulp complex

**FIGURE 7:**
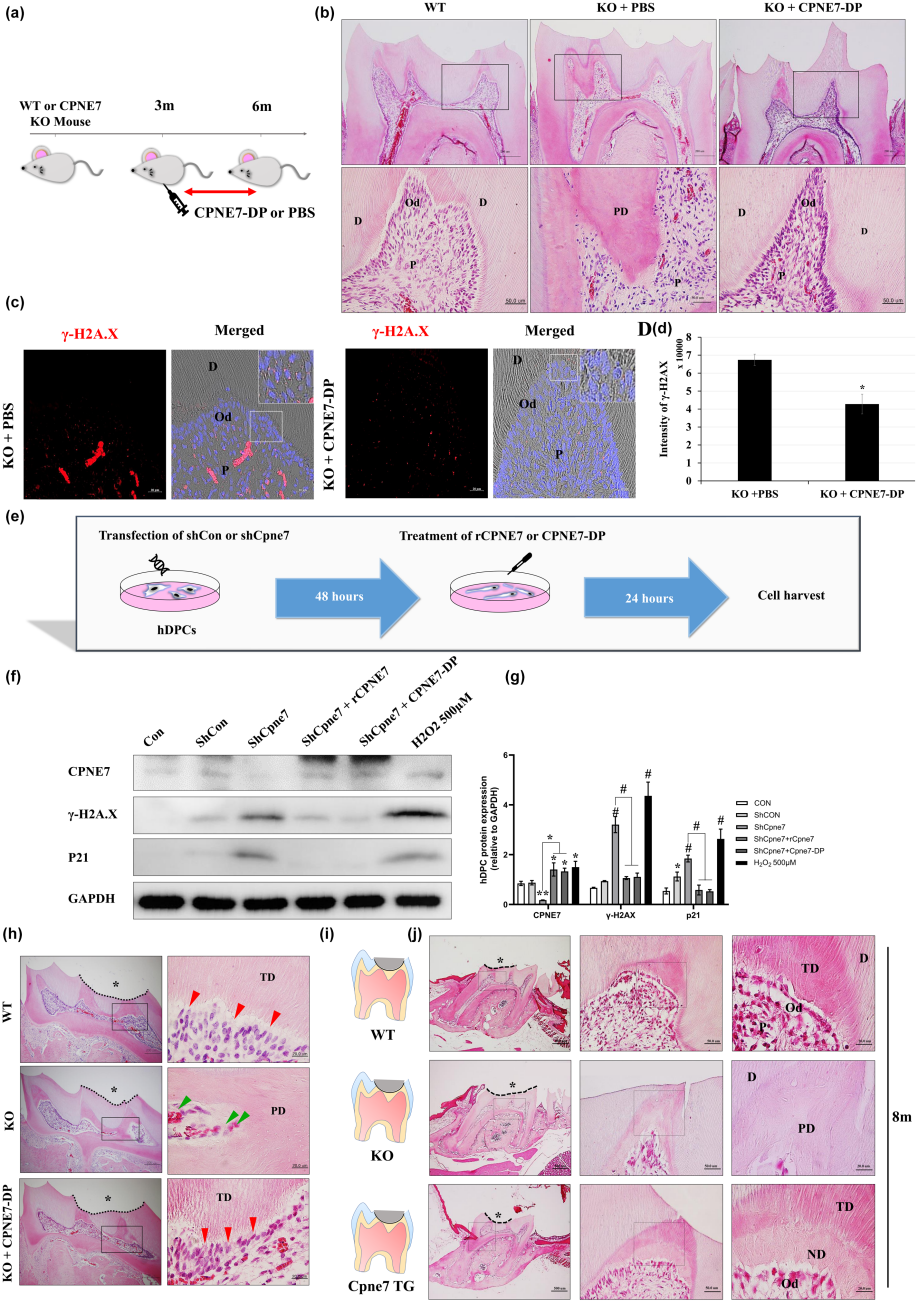
CPNE7‐derived functional peptide rescues the pathologic dentin‐pulp complex phenotypes in *Cpne*7^−/−^ mice molar. (a) Schematic diagram of the intraperitoneal CPNE7‐derived functional peptide (CPNE7‐DP) injection into WT and *Cpne*7^−/−^ mice. (b) Histological analysis of dental pulp responses with or without intraperitoneal CPNE7‐DP injection in WT and *Cpne7* ^−/−^ mice 3 months after, by H&E staining. Scale bars: 200 μm. Boxed areas are shown at higher magnification. Scale bars: 50 μm. D, dentin; P, pulp; PD, pathologic dentin. (c) Representative immunofluorescence images of γ‐H2AX (red) with or without intraperitoneal CPNE7‐DP injection into 6‐month‐old *Cpne7* ^−/−^ mice molar. DAPI (blue) was counterstained to indicate the nucleus. Scale bars: 20 μm. Boxed areas were shown at higher magnification. D, Dentin; Od, Odontoblasts; P, Dental pulp. (d) Semi‐quantification of γ‐H2AX (red) signal was analyzed. (e) Schematic diagram of DNA damage rescue model in shCon or shCpne7‐transfected hDPCs. (f, g) CPNE7, γ‐H2AX, and P21 protein levels were evaluated by western blot analysis and semi‐quantified. (h) Histological analysis of dental pulp responses at the cavity preparation sites with or without CPNE7‐DP treatment in WT and *Cpne7* ^−/−^ mice 3 weeks after H&E staining. *, Defected area; D, Dentin; Od, Odontoblasts; P, Dental pulp; PD, Pathologic dentin; green arrowhead, entrapped cells; red arrow, odontoblast‐like cells. (i) Schematic diagram of the indirect pulp capping model. Defect areas were restored with glass ionomer (GI) cement in WT, *Cpne7* ^−/−^, and *Cpne7* TG mice at 8 months. (j) Histological analysis of the defected areas after 4 weeks was performed by H&E staining. Scale bars: 500 μm. Boxed areas are shown at higher magnification. Scale bars: 50 μm, 20 μm. *, Defected area; D, Dentin; Od, Odontoblasts; P, Dental pulp; PD, Pathologic dentin; ND, Newly‐formed physiologic dentin. *p < 0.05 and #*p* < 0.0001 vs. Control.

In our previous study, we synthesized a CPNE7‐derived peptide (CPNE7‐DP) that replicates the functions of CPNE7 protein (Lee et al., 2020). In order to examine whether treating CPNE7‐DP can restore the structural and cellular alterations in *Cpne7* ^−/−^ pulp, we performed intraperitoneal injection of CPNE7‐DP or 1×PBS (control) into 3‐month‐old *Cpne7* ^−/−^ mice every 2 days for a total of 3 months and implemented histological analysis (Figure 7a). Interestingly, the disruption of odontoblast alignment and the evident decrease in dental pulp cell population were rescued in the CPNE7‐DP injected group, and even a denser cell population was observed, compared to WT (Figure 7b). The rescued phenotypes showed no difference between male and female mice histologically (data not shown). In addition, CPNE7‐DP injected molars showed less DNA damage accumulation in dental pulp cells than the 1×PBS injected molars (Figure 7c,d). To assess whether intraperitoneally injected CPNE7‐DP reaches the dental pulp environment through blood circulation, [^14^C]‐labeled CPNE7‐DP after intravenous administration was analyzed in rats in vivo (Figure S12). The tissue distribution of CPNE7‐DP and its pharmacokinetics were evaluated. When 2.4 mg/kg of [^14^C]‐labeled CPNE7‐DP was administered once, the maximum concentration was reached in most tissues within 10 min and widely distributed throughout the body. The distribution of [^14^C]‐labeled CPNE7‐DP was highly detected in the order of pancreas, adrenal gland, and bone marrow 10 min after injection, and a comparatively small amount was also detected in the tooth.

Next, to identify the effects of injected CPNE7‐DP at a cellular level, we analyzed DNA damage accumulation in hDPCs treated with rCPNE7 or CPNE7‐DP after ShCpne7 transfection (Figure 7e). As demonstrated earlier, the expression level of γ‐H2AX was significantly increased in the *Cpne7*‐deleted group compared to the control (Figure 7f,g). Treating 500 μM H_2_O_2_ to the hDPCs induced DNA damage and cell cycle arrest, demonstrated by upregulated expressions of γ‐H2AX and P21, respectively (Figure S13). When rCPNE7 or CPNE7‐DP was treated after ShCpne7 transfection, the expression level of γ‐H2AX was evidently diminished (Figure 7f,g). Moreover, the expression level of P21, which contributes to the senescence induction by pausing the cell cycle until damaged DNA gets repaired, was especially increased in the CPNE7‐DP treated group (Figure 7g).

We next assessed the effects of topical application of CPNE7‐DP onto the dentinal defects. Pathologic dentin‐pulp environment was observed in the *Cpne7* ^−/−^ mice, but a robust dental‐pulp environment was confirmed in the group to which CPNE7‐DP was applied topically (Figure 7h). Next, to investigate the response of dental pulp cells to strong external stimuli in *Cpne7* null or overexpressing conditions, we artificially generated dentinal defects in the mouse teeth by mechanical drilling (Figure 7i). As a physiological defense mechanism, a small amount of tertiary dentin was formed under the defect in WT mice (Figure 7j). On the other hand, pathologic tertiary dentin with cell entrapments and disorganized odontoblast layer was formed in *Cpne7* ^−/−^ mice (Figure 7j). In the *Cpne7* TG mice, the newly formed physiologic dentin retained a tubular structure with a typical odontoblast alignment below, suggesting that continuous expression of Cpne7 contributes to maintaining the physiological functions of odontoblasts. These results suggest that it can be an innovative medicine for maintaining a healthy dental‐pulp environment.

## 3 DISCUSSION

An aging oral environment exhibits atrophies of both structural and functional features, including wear of enamel and dentin, higher incidence of root caries, loss of periodontal attachment, and reduced healing capacity. An increasing number of elderly patients will retain their natural teeth in years to come, but age‐related diseases have been under‐explored in dentistry. The ability of dental pulp to repair itself gradually declines with age, increasing the risk of tooth vitality loss (Iezzi et al., 2019). Necrotic pulp tissue should be disinfected promptly through root canal treatment, but age‐related dystrophic calcification in dental pulp challenges the completeness of the clinical procedure (Negishi et al., 2005). It is therefore possible for the damage to extend to periapical and peri‐radicular tissues, affecting the whole periodontium. Here, we report that *Cpne7* ^−/−^ teeth show features of the aged dental pulp, including a smaller pulp space followed by extensive calcification, low cellularity, reduced blood supply, and etc (Maeda, 2020). It was shown that pulp cell and odontoblast populations began to decline rapidly in the pulp of 6‐month‐old *Cpne7* ^−/−^ mice. Attrition of molars also occurred as part of natural aging processes in both WT and *Cpne7* ^−/−^ mice. In response to the physiological irritation such as masticatory force, typical reactionary tertiary dentin with tubular architecture formed in the pulp of WT mice, whereas abnormal and irregular calcified tissue known as pathologic dentin developed in *Cpne7* ^−/−^ mice. As cell survival was severely impaired in the *Cpne7* ^−/−^ mouse pulp, the round‐shaped cells embedded in the newly formed hard tissue were mostly dead.

Previously, we reported that Cpne7 induces odontoblast differentiation (Oh et al., 2015). In the present study, we further demonstrated that Cpne7 specifically induces the differentiation of lineage‐specific odontoblast progenitors, such as dental pulp stem cells, into odontoblasts. Other cells in the periodontium including GFs and PDLSCs did not undergo odontoblastic differentiation upon rCPNE7 treatment, nor did adipose stem cells or BMSCs. A sub‐odontoblast layer in the dental pulp is populated with progenitor cells that are recruited to replace injured odontoblasts (Sloan & Smith, 2007). In the absence of *Cpne7*, the progenitor cells in the sub‐odontoblast layer failed to differentiate toward mature odontoblasts. Overall, the loss of odontoblast population that can physiologically respond to external stimuli, in addition to the inability to restore the loss by new odontoblast differentiation resulted in the pathologic tertiary dentinogenesis in *Cpne7* ^−/−^ molars.

Cellular senescence generally refers to cellular stress responses provoked by endogenous and exogenous insults (Di Micco et al., 2021). In proliferation‐competent cells, senescence is eminently characterized by irreversible cell cycle arrest (Hayflick & Moorhead, 1961). In terminally differentiated post‐mitotic cells, accumulation of cellular stresses may turn the cells into a non‐dividing, yet chronically pathologic state (von Zglinicki et al., 2021). As a function of maintaining tissue homeostasis, senescence ensures that damaged and aged dysfunctional cells are suppressed; however, aberrant accumulation of senescent cells may cause aging and age‐related pathologies (McHugh & Gil, 2018). Hutchinson‐Gilford progeria syndrome is a well‐documented genetic disorder demonstrating dramatically accelerated aging of major organs and tissues of the body including teeth, which exhibit similar characteristics to those of *Cpne7* ^−/−^ mouse (Choi et al., 2018; Merideth et al., 2008). In the present study, 12‐month‐old *Cpne7* ^−/−^ mouse molar showed a similar histological phenotype to 23‐month‐old WT mouse molar, suggesting that the loss of Cpne7 function accelerates aging of the dentin‐pulp complex. Promotion of cellular senescence was evidenced by more intense SA‐β gal activity in *Cpne7* ^−/−^ pulp, while absence of *Cpne7* in vitro showed increased SA‐β gal activity during the odontoblastic differentiation of hDPCs. Moreover, a tumor‐suppressor gene *p21*, which is highly expressed in senescent cells, was upregulated by *Cpne7* loss. Interestingly, *Cpne7*‐overexpressing TG mouse demonstrated delayed aging of the dentin‐pulp complex at 23 months, as recombinant CPNE7 treatment in vitro significantly alleviated ROS‐induced cellular senescence in hDPCs.

Aged cells generally exhibit an elevation of intracellular ROS levels and an accumulation of DNA damage (Wang et al., 2009). In this study, we observed a high level of ROS in *Cpne7* ^−/−^ pulp. Oxidative stress is a major cause of DNA damage, while DNA damage itself can induce ROS generation that mediates cell death or senescence (Kang et al., 2012; Li et al., 2006; Rowe et al., 2008). High concentrations of ROS disrupt cell viability, but sublethal doses generate oxidative stress that leads to what is commonly known as stress‐induced premature senescence (SIPS) (Toussaint et al., 2000). Double‐strand break is known as one of the most detrimental forms of DNA damage, the proper repair of which is essential for genomic stability. Faultily repaired double‐strand breaks can compromise the self‐renewal and differentiation capacity of dental pulp stem cells (Feng et al., 2014). A phosphorylated form of histone H2AX (γ‐H2AX) indicates the site of DNA damage, since its phosphorylation occurs shortly after DNA double‐strand breaks (Mah et al., 2010). Loss of *Cpne7* resulted in DNA damage accumulation in dental pulp, indicated by increased γ‐H2AX signals. Here, we first demonstrated that Cpne7 is recruited to the sites of DNA damage, and reduces H2AX phosphorylation and DNA fragmentation induced by oxidative stress. Knocking down *Cpne7* significantly downregulates *Chd6*, an oxidative DNA damage response factor, and *Timeless*, which functions during homologous recombination repair (Moore et al., 2019; Xie et al., 2015). Eukaryotic DNA is packaged with histone proteins into a complex called chromatin, which hinders the factors involved in DNA repair from accessing its substrate. The chromatin at the site of double‐strand break undergoes PARylation to recruit active complexes essential for subsequent remodeling (Stadler & Richly, 2017). The RNA sequencing data from the present study imply that Cpne7 is functionally related to chromatin remodeling, and upregulates genes coding for histones and post‐translational modifications, yet, whether Cpne7 affects DNA damage repair through modulating chromatin remodelers should be investigated further. Taken together, Cpne7 may contribute to genomic stability and functional integrity of dental pulp cells through participating in early DNA damage response, the exact mechanism of which should be elucidated in the future.

It is also noteworthy that treating CPNE7‐derived functional peptide (CPNE7‐DP) had a preventive effect on cellular damage caused by oxidative stress in dental pulp cells. Moreover, injection of CPNE7‐DP rescued the premature aging phenotype of *Cpne7* ^−/−^ mouse pulp. Unlike topical application onto the exposed dentin, intraperitoneal injection enables a drug to enter the systemic circulation and to affect the target tissue more broadly. The pulp cell population including a palisade layer of odontoblasts were restored to the level of the WT after 3 months of intraperitoneal injection of CPNE7‐DP. It can be suggested that CPNE7‐DP repairs the compromised cell survival in *Cpne*7^−/−^ mouse pulp, possibly by promoting proliferation and odontogenic differentiation of the dental pulp stem cells. Possible clinical applications of CPNE7‐DP in treating dentinal hypersensitivity and arresting dental caries have been introduced previously (Gug et al., 2022; Lee, Hwang, et al., 2022; Park et al., 2019). CPNE7 and its derivative functional peptide can penetrate the dentinal tubules to affect the underlying odontoblasts and pulp cells, resulting in mineralization activity. Here, the more fundamental aspect of its therapeutic effects can be proposed, as Cpne7 was proven to play an important role in maintaining appropriate functions of dental pulp cells, including odontoblasts, by preventing cellular damages caused by oxidative stress.

## 4 METHODS

### 4.1 Cell culture

The hDPCs were obtained from the Seoul National University Dental Hospital, and the experimental protocol was accepted by the Institutional Review Board of the Seoul National University (S‐D20140007). Informed consent was obtained from patients. Human DPCs were isolated and used in an in vitro experiment as described previously (Jung et al., 2011). Human DPCs were cultured in minimum essential medium α (Gibco BRL, 12571063) supplemented with 10% heat‐inactivated fetal bovine serum (FBS; Gibco BRL, 16000044) and antibiotic‐antimycotic (Gibco BRL, 15240062) at 37°C in an atmosphere with 5% CO_2_. The cells were then treated with recombinant CPNE7 protein (rCPNE7, 100 ng/mL; Origene, TP306428) or CPNE7‐derived peptide (CPNE7‐DP, 10 μg/mL). After oxidative stress‐induced DNA damage using Hydrogen Peroxide Solution (H_2_O_2_; Merck, H1009) rCPNE7 was treated after replacement with a new culture medium (α‐MEM).

### 4.2 Plasmids and transient transfection

Expression vectors encoding full‐length human *Cpne7*, green fluorescent protein (GFP)‐CPNE7 (NM_153636), and Recombinant CPNE7 (rCPNE7; Oriegene, NP_705900) were purchased from Origene (Rockville, MD). Control shRNA (TR20003) and shRNA targeting *Cpne7* (TR305242) were purchased from Origene (Rockville, MD). The cells were transiently transfected using Lipofectamine™ LTX Reagent with PLUS™ Reagent (Thermofisher Scientific, 15338100) with the constructs.

### 4.3 Live cell imaging

The morphologic change of hDPCs treated with or without rCPNE7 was measured by live‐cell imaging (*n* = 3, each group). After being treated with rCPNE7 for 24 h, the cells were differentiated for 7 days and scanned for 2 days. Bright‐field images of the cells were acquired using a confocal laser scanning microscopy system (LSM980, Zeiss, Germany). The scanning rate was 1.12 s per scan for 16‐bit images of 1024 × 1024 pixels in size. Timelapse images collected from a single Z plane were recorded at 8‐min intervals.

Human DPCs were transfected with 2 μg *Cpne7* expression construct (GFP‐tagged; Origene, NM_153636) for 48 h. DNA damage tracks were induced in live cells (kept at 37°C in a humidified environment at 5% CO_2_) using a 405 nm 5 mW self‐aligning solid‐state diode laser (15 μm/s, 30% power) projected through an EC Plan‐Neofluor 40× objective, via a Zeiss PALM MicroBeam laser microdissection module on a Zeiss Axio Observer Z1 platform (*n* = 3, each group). An AxioCam MRm Rev.3 camera was used to capture images. Laser irradiation was controlled by RoboSoftware 4.5. Zen Pro (Zeiss) was used for acquisition and as an analysis software. CZI image files captured every 60 s for the indicated times were subsequently analyzed by Zen image processing software.

Human DPCs were stained with DCFDA‐cellular ROS assay kit (Abcam, ab113851) for 40 min at 37°C in a humidified environment at 5% CO_2_. Then, live cell imaging was performed with a filter set appropriate for fluorescein (FITC) (*n* = 3, each group). Low light conditions were maintained to avoid photobleaching and photo‐oxidation of DCF.

### 4.4 Mouse subcutaneous ex vivo transplantation

The hDPCs, ADSCs, BMSCs, GFs, and PDLSCs (2 × 10^6^) were mixed with 100 mg of hydroxyapatite/tricalcium phosphate ceramic powder (Zimmer) alone or with rCPNE7 (100 ng/mL) in an 0.5% fibrin gel. The poly ε‐caprolactone (PCL: Mw 80,000, Sigma–Aldrich, Oakville, ON, Canada) was wrapped around the mixed cells and transplanted subcutaneously into immunocompromised mice (NIHbg‐nu‐xid; Harlan Laboratories) for 6 weeks (*n* = 3, each group).

### 4.5 Immunocytochemistry and immunofluorescence

Human DPCs and tissue sections were treated with phosphate‐buffered saline (PBS) and 0.5% Triton X‐100 for permeabilization. After washing and blocking, cells and tissue samples were incubated overnight at 4°C with γ‐H2AX (1:100, Cell Signaling Technology, #9718) and CPNE7 (1:100) in blocking buffer (PBS and 2% bovine serum albumin). Subsequently, FITC‐conjugated anti‐rabbit secondary antibody (1:500) or Cy3‐conjugated anti‐rabbit (1:500) was applied. Cells were visualized using confocal microscopy (LSM980, Zeiss, Germany). The chromosomal DNA in the nucleus was stained with 4′,6‐diamidino‐2‐phenylindole (DAPI). The microtubules were detected using α‐TUBULIN antibodies (1:1000; Santa Cruz), and F‐ACTIN was stained with Alexa Fluor 488 or rhodamine‐conjugated phalloidin (Invitrogen). Flag‐tagged rCPNE7 was detected using Flag‐antibodies (1:500; Sigma–Aldrich), and TAU was detected by TAU antibodies (1:100; CUSABIO). Immunofluorescence staining of TAU, F‐ACTIN, and α‐TUBULIN was analyzed using the IMARIS cell imaging software (Bitplane) (*n* = 3, each group).

### 4.6 Immunohistochemistry

Briefly, sections were incubated overnight at 4°C with rabbit DSP antibody (1:200); immunization of rabbit with the synthetic peptides NH2‐GNKSIITKESGKLSGS‐COOH (amino acid residues 372 ~ 387 of DSP) and BSP antibody (1:200) in 5% skim milk/PBS, pH 7.4. Negative control sections were incubated with 5% skim milk/PBS. Sections were then incubated with a biotin‐labeled goat anti‐rabbit immunoglobulin G (IgG) (1:200; Vector Labs) as the secondary antibody and then washed and incubated with avidin‐biotin‐peroxidase complex (Vector Laboratories Inc., Burlingame, USA). Peroxidase was revealed by incubation with methanol containing 3% H_2_O_2_. Signals were converted using a diaminobenzidine kit (Vector Laboratories). Nuclei were stained with Hematoxylin (Vector Laboratory, Burlingame, CA, USA) (*n* = 3, each group).

### 4.7 Comet assay

Human DPCs were combined with Comet Agarose (Abcam, ab238544) at 1/10 ratio (v/v), mixed by pipetting, and immediately transferred onto the top of the Comer Agarose Base Layer Slide (Abcam, ab238544). Set horizontally, the slides were incubated at 4°C in the dark for 15 min, and placed in a small basin/container containing pre‐chilled Lysis Buffer (Abcam, ab238544). They were immersed in the buffer for 1 h at 4°C in the dark. After 1 h, aspiration of the Lysis Buffer from the container was done and replaced with a pre‐chilled Alkaline Solution (Abcam, ab238544), followed by electrophoresis chamber filled with the Alkaline Electrophoresis Solution. Voltage was applied to the chamber for 30 min at 1 volt/cm. The slides were then rinsed with pre‐chilled DI H_2_O and replaced with cold 70% Ethanol for 5 min. The slide was removed from the 70% Ethanol and allowed to air dry. Once the slide was completely dry, staining with Vista Green DNA Dye (Abcam, ab238544) was done at room temperature for 15 min. Cells were visualized using confocal microscopy (LSM980, Zeiss, Germany) (*n* = 3, each group).

### 4.8 Senescence β‐galactosidase (SA‐β gal) staining

Human DPCs were fixated with Fixative Solution (Cell Signaling Technology, #9860) at room temperature for 15 min. Rinsed twice with PBS, the cells were then stained with β‐Galactosidase Staining Solution (Cell Signaling Technology, #9860) at 37°C overnight in a dry incubator (no CO_2_). Cells were observed using light microscopy (Primovert, Zeiss, Germany) (*n* = 3, each group).

### 4.9 Cell cytotoxicity assay

Human DPCs were seeded in 96‐well plates at 3 × 10^3^ cells/well and incubated for 24 h in α‐MEM. The starting medium was replaced with medium only (control group), with H_2_O_2_ 50 μM, 100 μM, 200 μM, 500 μM, 1 mM, or 2 mM (experimental groups) for 24 h. To evaluate cytotoxicity, WST‐8 (Medifab, B1007) was added to the control and the experimental groups after 4 h. Cell cytotoxicity was measured using a luminometer (FLUOStar OPTIMA; BMC Laboratory).

### 4.10 Peptide synthesis

CPNE7‐DP consists of 10 amino acids from 344 to 353 fragment (KYKQKRRSYK) of the hCPNE7 protein. The peptide was synthesized using the Fmoc (9‐fluorenylmethoxycarbonyl)‐based solid phase method and characterized by Lugen Sci.Co., Ltd. (Bucheon, Korea). The purity of the peptide used in this study was greater than 97% as previously determined by high‐performance liquid chromatography (Lee et al., 2020).

### 4.11 Western blot analysis

Cellular proteins (30 μg) were separated by 10% or 15% sodium dodecyl sulfate‐polyacrylamide gel electrophoresis (SDS‐PAGE) and transferred to polyvinylidene fluoride (PVDF) membranes. The PVDF membranes were blocked for 1 h with 5% nonfat dry milk in Tris‐buffered saline containing 0.1% Tween 20 (TBS‐T) and incubated overnight at 4°C with the primary antibody diluted in TBS‐T (1:5000). After washing, membranes were incubated for 1 h with the appropriate secondary antibodies. Labeled protein bands were detected under an enhanced chemiluminescence system (Amersham Biosciences/GE Healthcare). All reactions were performed in triplicate. Semi‐quantitative analyses were performed using Image J software (National Institute of Health, MD, USA) (*n* = 3, each group).

### 4.12 Real‐Time polymerase chain reaction analysis

Total RNA was extracted with TRIzol® reagent (Invitrogen, Carlsbad, CA, USA) according to the manufacturer's instructions. Superscript III reverse transcriptase (Invitrogen) and oligo (dT) primers (New England Biolabs) were used to reverse transcribe total RNA (3 μg). The product (1 μL) was amplified by PCR using the following primer pairs: *hTimeless*, forward 5′‐CATCTAGCCCAGAAGAAGTG‐3′ and reverse 5′‐GAGACAGTCTTGGATCCGTA‐3′; *hChd6*, forward 5′‐ATGTACCTCTGCCTGACA TC‐3′ and reverse 5′‐GTCTGAGGTCCCATCTGTAA‐3′; *hCpne7*, forward 5′‐GGAGAC AAGGCCTCTAAAGT‐3′ and reverse 5′‐AGTACACCCTGTGGAACTTG‐3′; *hGapdh*, forward 5′‐ATCATCCCTGCCTCTACTG‐3′ and reverse 5′‐TTG AAGTCAGAGGAGACCAC‐3′. ABI PRISM 7500 sequence detection system (Applied Biosystems, Carlsbad, CA, USA) and  SYBR Green PCR Master Mix (Takara Bio, Shiga, Japan) were used according to the manufacturer's instructions. Following condition was set: 40 cycles of 95°C for 1 min, 94°C for 15 s, and 60°C for 34 s. Housekeeping gene *Gapdh* was used to normalize PCR products. All reactions were performed in triplicate. Relative changes in gene expression were calculated using the comparative threshold cycle (Ct) method (*n* = 3, each group).

### 4.13 Generation of Copine VII‐null mice and K14 promoter Copine VII‐transgenic mice

*Cpne7* gene was cloned from a strain 129/SVEV mouse genomic DNA. *Cpne7*‐null mice were generated with standard gene targeting methods. The targeting construct (Figure S2a) included an *EGFP* coding region (*EGFR*) and Neomycin resistance cassette (*NeoR*), in place of the coding sequence for *Cpne7* (exon 5–7). The construct was electroporated into the CMTI‐1 ES cell line derived from mouse strain 129/SVEV. After electroporation, G418/ganciclovir‐resistant colonies were screened by PCR by using the primers depicted in Figure S2b (F1, 5‐CAATATGGGATCGGCCATTGAAC‐3; F2, 5‐GCCTTCTTGACGAGTTCTTCTGA‐3; R1, 5‐ACAGACAGACAAACTCATGTTGG‐3; R2, 5‐CCTGTAGGGAACGGAGGGGTG‐3). The targeted cells yielded a 3.45‐kb, 2.67‐kb, and 2.40‐kb PCR product, according to the primer sets, respectively. To generate transgenic mice that overexpress *Cpne7* in epithelial and endothelial cells, *Cpne7* cDNA was inserted into the rodent expression vector (NM_170684, Origene) by replacing the K14 promoter at the CMV promoter site, and Rabbit β‐globin intron at the VP 1.5 primer and T7 promoter. Male chimeras carrying the disrupted allele were mated with wild‐type C57BL/6N strain females, and their heterozygous progeny were mated to obtain *Cpne7*‐null mice or *Cpne7* TG mice with a genetically uniform background. To genotype the mice, DNA was isolated from a small piece of tail tissue, and PCR was performed. All experiments were approved by the Institutional Animal Care and Use Committee of Seoul National University (Seoul, Republic of Korea).

### 4.14 Micro‐computed tomography analysis

Mice maxilla and mandible (*n* = 3, each group) were dissected, fixed in 4% paraformaldehyde for 24 h at 4°C, and washed with PBS for 24 h at 4°C. The samples were scanned using a Bruker SkyScan1172 micro–computed tomography (μCT) scanner. For visualization and analysis, DataViewer, CTAn, CTVol, and CTVox software programs (Bruker microCT) were used. Two‐dimensional images were obtained from μCT cross‐sectional images of the mandible and maxilla. Molars and incisors were evaluated for their mineral formation using the DataViewer program. Three‐dimensional reconstructions were performed to confirm the exterior phenotypes of the teeth and the alveolar bone and to verify the formation of artificial tooth cavity. For the assessment of tissue mineral content, a region of interest (ROI) of X = 1.00 mm, Y = 1.15 mm, and Z = 1.46 mm was set as standard for all samples, and the mineral analysis was performed. The ROI comprised the crown of primary molar above the level of alveolar bone. From the μCT scans of 12‐month‐old WT and *Cpne7*‐deficient mandibles, primary molars were segmented out for measurement of tooth volume. Then from the primary molar segment, pulp space volume was measured using CTAn program. The mandible, primary molar, and pulp space were reconstructed in 3D using CTVol or CTVox program.

### 4.15 Histology and immunohistochemistry

The maxilla and mandible (*n* = 3, each group) were dissected from WT and mutant mice, and fixed immediately in ice‐cold 4% paraformaldehyde solution for 24 h at 4°C with gentle agitation. The samples were then washed with PBS for 24 h at 4°C. Decalcification was carried out in 0.5 M EDTA at room temperature with constant shaking. When the decalcification was complete, the samples were dehydrated in ethanol series and embedded in paraffin. Samples were then cut into 4‐μm‐thick sagittal sections using a Microtome (Leica). For immunohistochemistry, sections were deparaffinized and immersed in 0.6% H_2_O_2_/methanol for 20 min to quench the endogenous peroxidase activity. They were then pre‐incubated with 1% bovine serum albumin in PBS for 30 min and incubated with rabbit polyclonal BSP (1:200), DSP (1:200), DMP1 (1:100), or CPNE7 (1:200; Santa Cruz Biotechnology) antibodies overnight at 4°C. Next, sections were incubated for 1 h at room temperature with the secondary antibody and reacted with avidin‐biotin‐peroxidase complex (Vector Laboratories) in PBS for 30 min. After color development with 0.05% 3,3′‐diaminobenzidine tetrahydrochloride (Vector Laboratories), the sections were counterstained with hematoxylin. For histological analysis, deparaffinized sections were stained with hematoxylin and eosin (H&E) or masson's trichrome staining kit. For the number of cells under the tertiary dentin, hematoxylin‐stained nuclei were counted using the Count function in Adobe Photoshop (Adobe Photoshop CS6 for Windows, Adobe Systems, San Jose, CA, USA).

### 4.16 BrdU staining

To map the label‐retaining cells in dental pulp, four intraperitoneal (I.P.) injections (once a day) of BrdU (100 mg/kg of body weight; Sigma‐Aldrich) were given to WT and *Cpne7*‐deficient mice at 4‐month or 5‐month‐old age (*n* = 4, each group). The mice were sacrificed at 6‐month‐old age, and the mandibles were dissected and processed for paraffin embedding as described above. BrdU‐retaining cells were identified using a BrdU staining kit (Zymed Laboratory) according to the manufacturer's instructions and counterstained with hematoxylin. The brown color in the BrdU‐labeled cells was changed to a red color and graphically emphasized by using graphic software (Adobe Photoshop CS6 for Windows, Adobe Systems, San Jose, CA, USA). The labeling indices for BrdU‐immunopositive pulp and odontoblast cells were quantified using the ImageJ program.

### 4.17 Mouth tooth injury model

The 3‐month‐old mice were anesthetized with 1.25% Avertin (Tribromoethanol, 250 mg/kg body weight) solution in sterile water at rate of 10 mL/kg I.P. 100% stock Avertin solution was made by dissolving 5 g 2,2,2‐Tribromoethanol (sigma) in 5 mL 2‐methyl‐2‐butanol (sigma). To expose the dentine of the mouse superior molar teeth, a high‐speed handpiece with rounded carbide burr FG 1/4 was used. The teeth with dentin exposure were either treated with PBS or rCPNE7 (1 μg total per tooth in a buffer containing 25 mM Tris–HCl, 100 mM glycine, and 10% glycerol) and left at least 3 min for penetration through the dentinal tubules. The animals (*n* = 4, each group) were sacrificed 6 weeks after the cavity preparation.

### 4.18 Primary mouse pulp cell and apical bud cell culture

The mandibles (*n* = 10, each group) were removed from 1‐, 3‐, and 6‐month‐old WT and *Cpne7*‐deficient mice. Briefly, incisors were dissected out from the mandible and cracked longitudinally using a 27‐gauge needle on a 1‐mL syringe. The pulp tissues and labial cervical loop were removed gently with the needle or forceps, cut into several pieces, and placed on 60‐mm culture dishes (Nunc). The dissected tissues were soaked in 2% collagenase in 1× PBS for 3 h at 4°C in a low adherence plate and moved to cold Dulbecco's modified Eagle's medium/F12 (DMEM/F12) (Invitrogen). Lower end of the cervical loop was gently pulled using either a size 5 forceps or an insulin syringe (1 cc, 28 G ½); the mesenchyme fell apart while the epithelium remained intact. Cells were seeded in a six‐well plate at 1.6 × 10^4^ cell/mL in DESC media, which consists of DMEM/F12 supplemented with mouse EGF recombinant protein at a concentration of 20 ng/mL, FGF recombinant protein at a concentration of 25 ng/mL, 1× B27 supplement, and 1% antibiotic solution (penicillin, 100 U/mL, streptomycin, 50 μg/mL). The cells were cultured at 37°C in a humidified atmosphere containing 5% CO_2_ and passed down twice to be used in following experiments.

### 4.19 RNA‐sequencing

The isolated RNA (*n* = 3, each group) was shipped to Macrogen (Seoul, Korea) for the library preparation, sequencing, and analysis of data. The cDNA Library for sequencing was prepared using TruSeq Stranded mRNA LT Sample Prep Kit. Total RNA was randomly fragmented, converted to complementary DNA (cDNA), and amplified according to the manufacturer's protocol. The cDNA was then ligated to sequencing adapters. Sequencing was performed on Illumina's HiSeq2500 platform. HISAT2 was used for mapping trimmed reads to reference genome (UCSC mm10), and the transcript assembly was performed using StringTie. To identify differentially expressed genes (DEGs), the expression profile was calculated for each sample and normalized to FPKM. KEGG database and Gene Ontology were used to perform functional annotation and gene‐set enrichment analysis on DEGs for known gene annotation. Genes with |fc| 1.5 and independent *T*‐test raw *p*‐value <0.05 were counted as significant. For KEGG analysis, *p*‐value <0.05 was selected as significantly enriched.

### 4.20 Transmission electron microscopy

For transmission electron microscope analysis, mandibles (*n* = 3, each group) were dissected out and pre‐fixed in 2% paraformaldehyde, 2% glutaraldehyde fixative solution overnight at 4°C. The samples were then decalcified in 5% EDTA for 4 weeks or until decalcification is complete. For post‐fixation, the samples were trimmed to contain only molars and incubated in 1% OsO_4_ for 1 h at 4°C. The teeth were then dehydrated and embedded in Epon using standard procedures. One micrometer of semi‐thin sections was cut in the tertiary dentin area, then, thin gold‐colored sections were prepared with a diamond knife, placed on formvar‐coated grids and conventionally stained with uranyl acetate and lead citrate. Sections were observed and photographed with transmission electron microscope JEM1010, JEOL.

### 4.21 TUNEL assay

TUNEL kit (Roche Biochemicals, Basel, Switzerland) was used to detect apoptotic cells according to the manufacturer's instructions. Prior to enzymatic labeling, endogenous peroxidase activity was blocked in 3% H_2_O_2_. Enzymatically labeled cells were then incubated with 3,3‐diaminobenzidine tetrahydrochloride to yield a colored reaction product. For visualization, the sections (*n* = 3, each group) were incubated with diaminobenzidine tetrahydrochloride (DAB), and subsequently counter‐stained with hematoxylin or methyl green. Positive TUNEL signals were converted to red color using IHC Profiler of Image J software (National Institute of Health, USA).

### 4.22 Rat [C^14^] CPNE7‐DP intravenous administration studies

The pharmacokinetics of CPNE7‐DP was investigated after a single intravenous administration (IV) of CPNE7‐DP labeled with ^14^C ([C^14^] CPNE7‐DP) to male rats at a dose of 2.4 mg/kg (Drug Development Solutions Center, Sekisui Medical Co., Ltd, Japan). Dosing formulation was administered into the tail vein using a syringe equipped with an injection needle. The radioactivity in the plasma, cumulative excretion of radioactivity, quantitative whole‐body autoradiography (QWBA) analysis, and metabolite analysis were investigated in a time‐dependent manner (Drug Development Solutions Center, Sekisui Medical Co., Ltd, Japan). HPLC with RAD and LSC was used. For radioactivity assay in the HPLC fractions, the HPLC eluate was fractionated at a constant interval of 23 s, and the radioactivity was measured using LSC. HPLC eluate over the run time was measured to calculate the recovery of radioactivity in the HPLC analysis (column recovery, %).

### 4.23 Statistical analysis

All data were expressed as the mean ± standard deviation of triplicate experiments. Statistical significance was analyzed using a non‐parametric Mann–Whitney *U* test for comparisons between two groups, and one‐way analysis of variance (ANOVA) with Bonferroni correction for comparisons between more than two groups by the SPSS software version 25. *p* values <0.05 were considered statistically significant.

## AUTHOR CONTRIBUTIONS

All authors contributed to the study conception, acquisition, and analysis of the data, and were involved in revising the article critically. Study design and manuscript writing: Yoon Seon Lee, Yeoung‐Hyun Park, Gene Lee and Joo‐Cheol Park.

## CONFLICT OF INTEREST STATEMENT

None declared.

## Supporting information


Appendix S1

Video S1–S3

## Data Availability

All data reported in the paper are included in the manuscript or are available in the Supplementary Information.
