# Supplementary material for: Cpne7 deficiency induces cellular senescence and premature aging of dental pulp
Source: Aging Cell. 2023 Dec 17;23(3):e14061. doi: 10.1111/acel.14061 (PMC10928576; doi:10.1111/acel.14061)
Supplement: Supplementary file 1 — Appendix S1 [file ACEL-23-e14061-s002.docx]

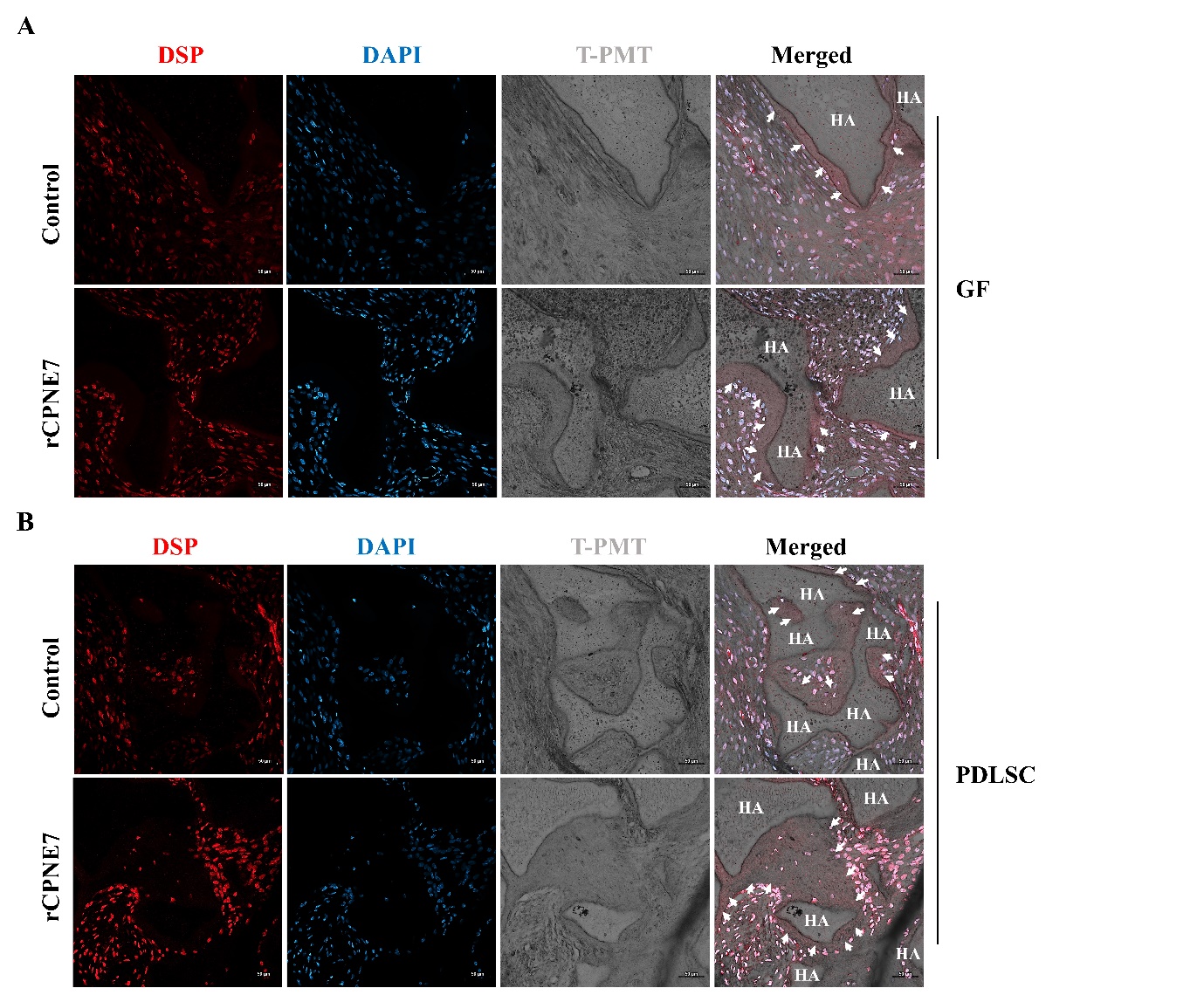


**Figure S1. (A)** The gingival fibroblasts (GFs) or **(B)** periodontal ligament stem cells (PDLSCs) were mixed with 100㎎ of hydroxyapatite/tricalcium phosphate (HA) particles alone (control) or with rCPNE7 in a poly ε-caprolactone (PCL) spaces and transplanted subcutaneously into immunocompromised mice for 6 weeks. White arrows indicate newly formed mineralized tissues. Representative immunofluorescence images of DSP (red) were observed (n=3). DAPI (blue) was counterstained to indicate the nucleus. Scale bars: 50㎛.


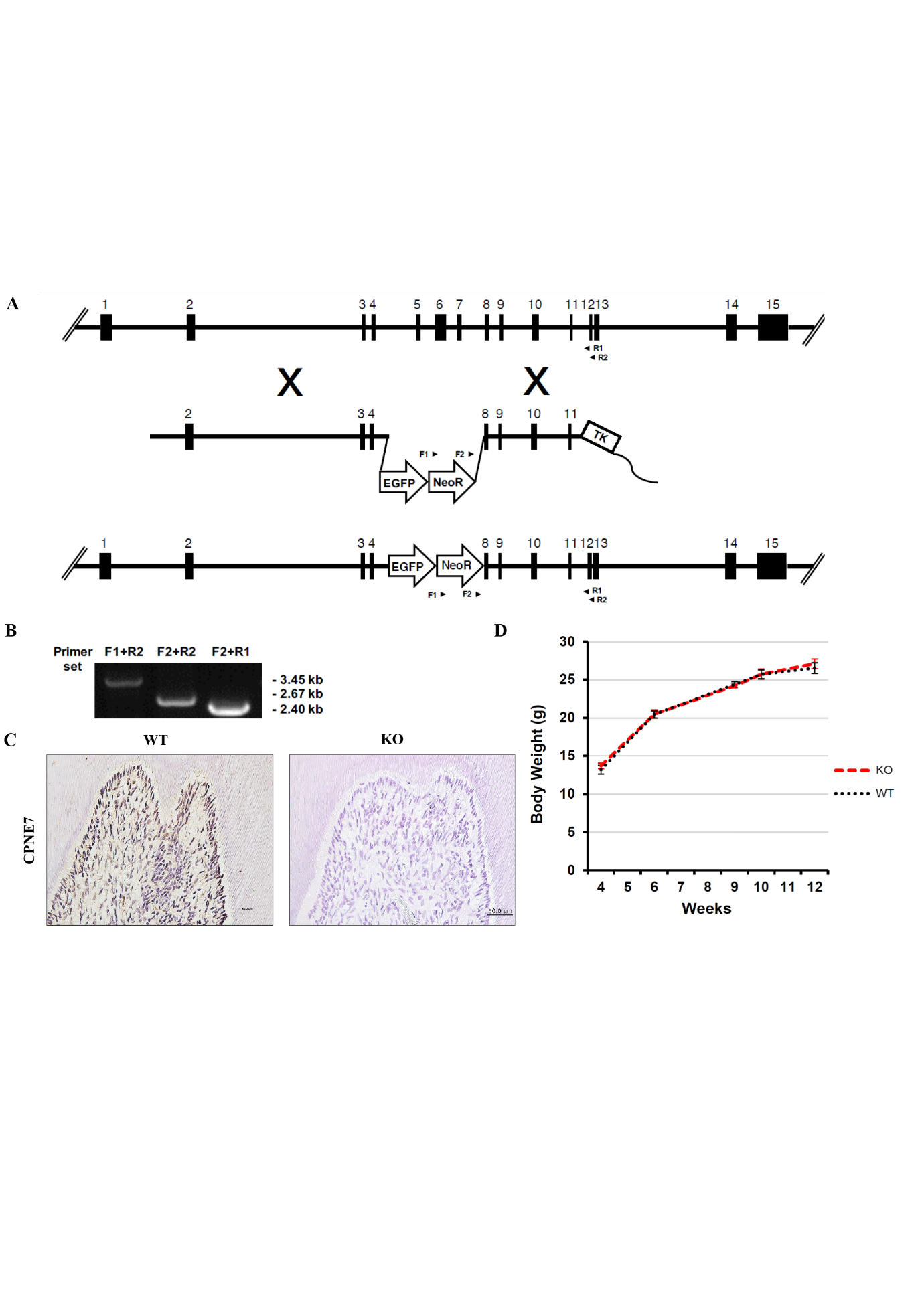


**Figure S2. Generation of Cpne7-null mice by targeted disruption of Cpne7 gene. (A)** Endogenous Cpne7 gene locus. The black box depicts the coding region. Targeting construct. NeoR, Neomycin resistance cassette; TK, thymidine kinase. Targeted allele after homologous recombination. Arrowheads and names indicate the PCR primers. **(B)** Detection of the targeted allele by PCR. The PCR primer sets amplify a 3.45-kb, 2.67-kb, and 2.40-kb PCR product respectively from the targeted allele. **(C)** CPNE7 protein expression in WT and KO mice pulps at 3 months. **(D)** Body weight change profiles of WT and KO mice.

**
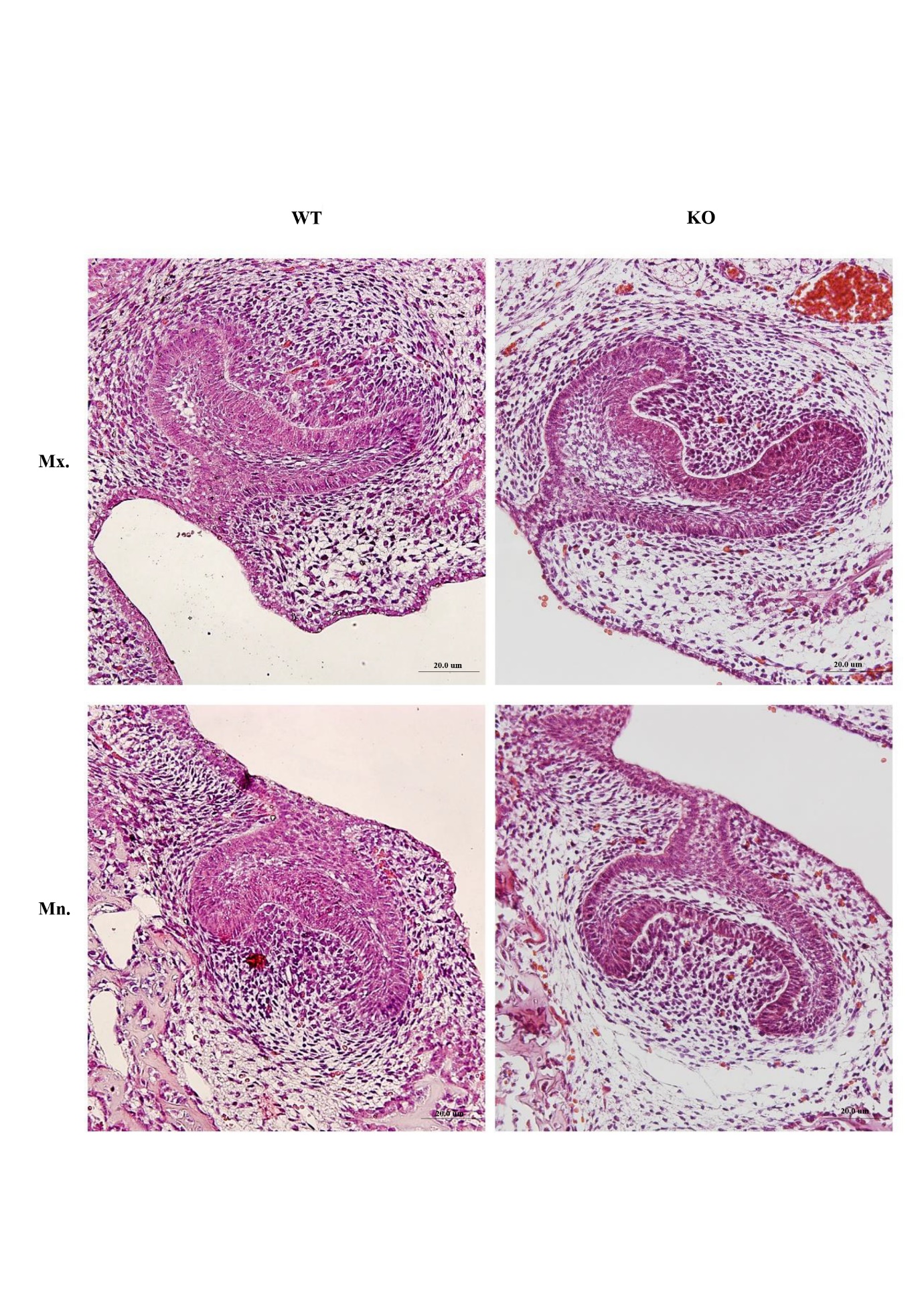
**

**Figure S3. Developing tooth germs of WT and Cpne7^-/-^ mice at E18.** Histological analysis of mandibular and maxillary first molars of WT and Cpne7^-/-^ mice at E18 by H&E staining. Scale bars: 20㎛.

**
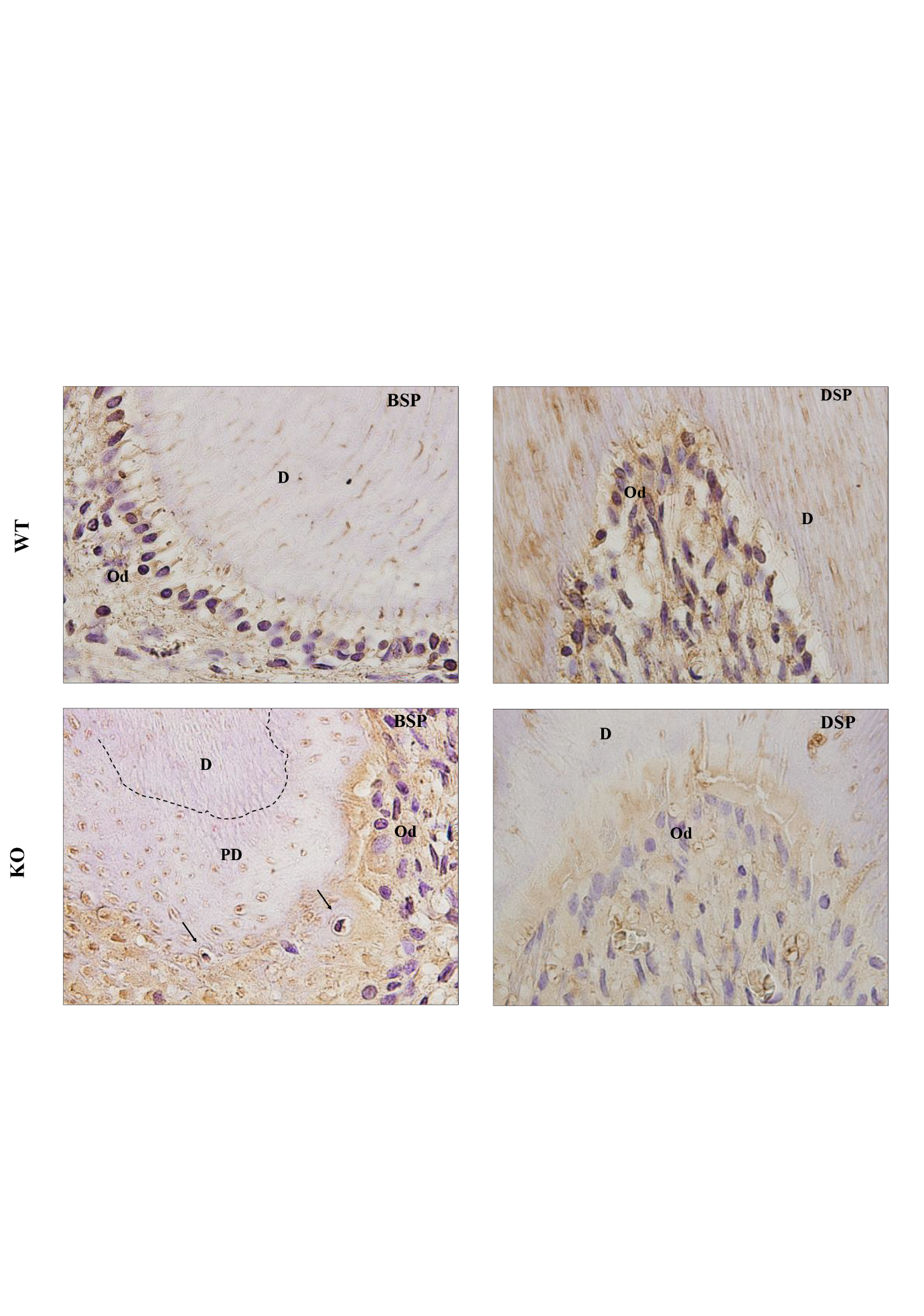
**

**Figure S4. Immunohistochemical analysis of molars of 6-month-old WT and Cpne7^-/-^ mice.** Protein expressions of BSP and DSP were detected by immunohistochemistry. D, dentin; Od, odontoblasts; PD, pathologic dentin.


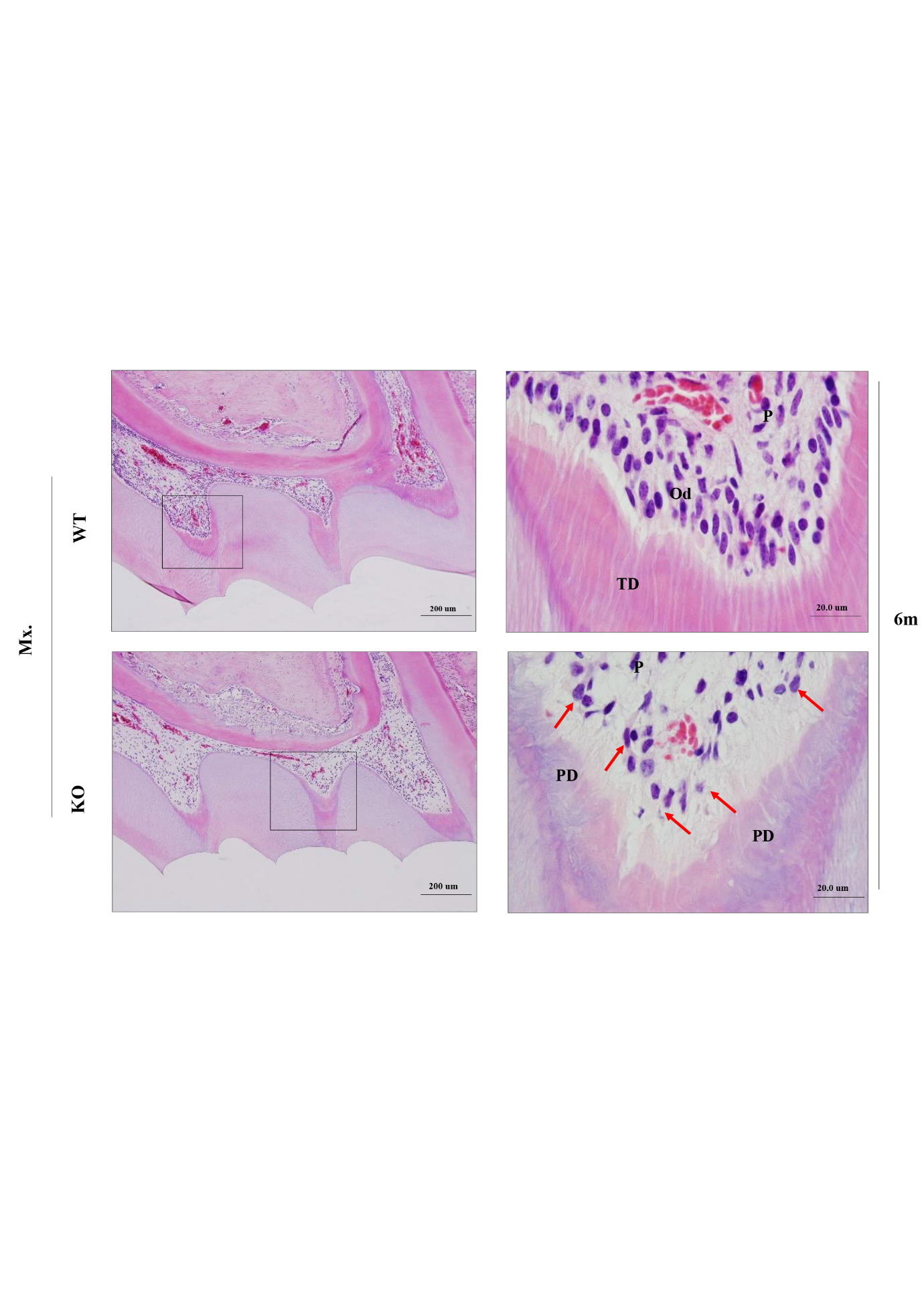


**Figure S5. Cpne7 deletion leads to pathologic changes in the mouse molar pulp environment.** Histological analysis of maxillary first molars of WT and Cpne7^-/-^ mice at 6 months by H&E staining. Scale bars: 200㎛. Boxed areas are shown at higher magnification. Scale bars: 20㎛. D, Dentin; Od, Odontoblasts; P, Dental pulp; PD, Pathologic dentin; Red arrow, cells getting entrapped.


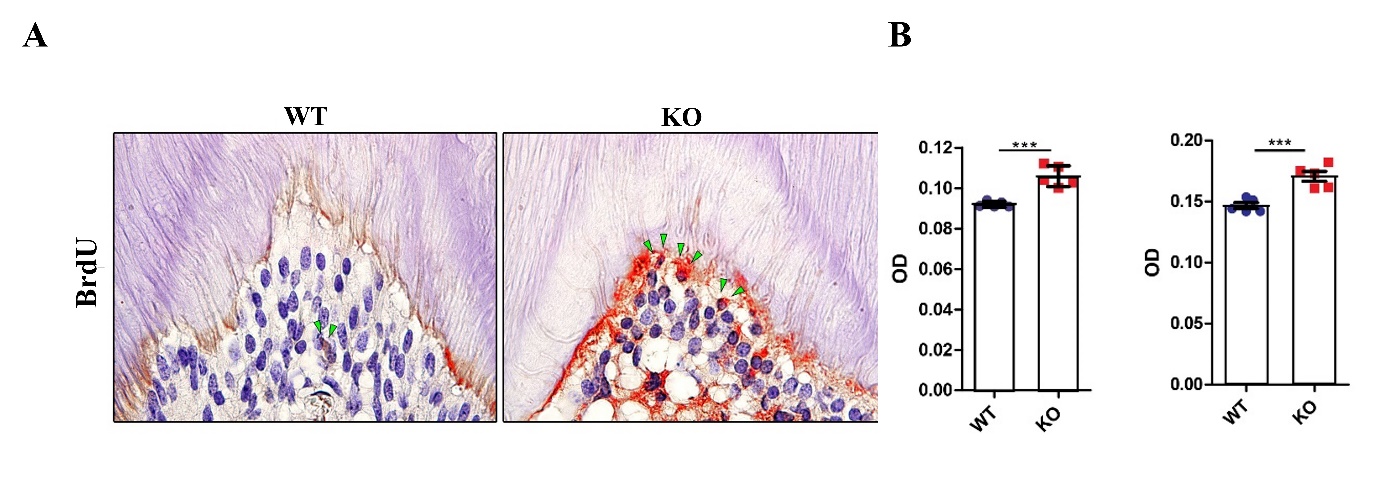


**Figure S6. (A)** Detection and semi-quantification of BrdU-positive dental pulp cells (green arrowhead) in WT and Cpne7^-/-^ mice at 6 months. **(B)** Quantitative analysis of BrdU-positive dental pulp cells in WT and Cpne7^-/-^ mice molars. All values represented the mean ± SD of triplicate experiments. ***P<0.001 vs Control.


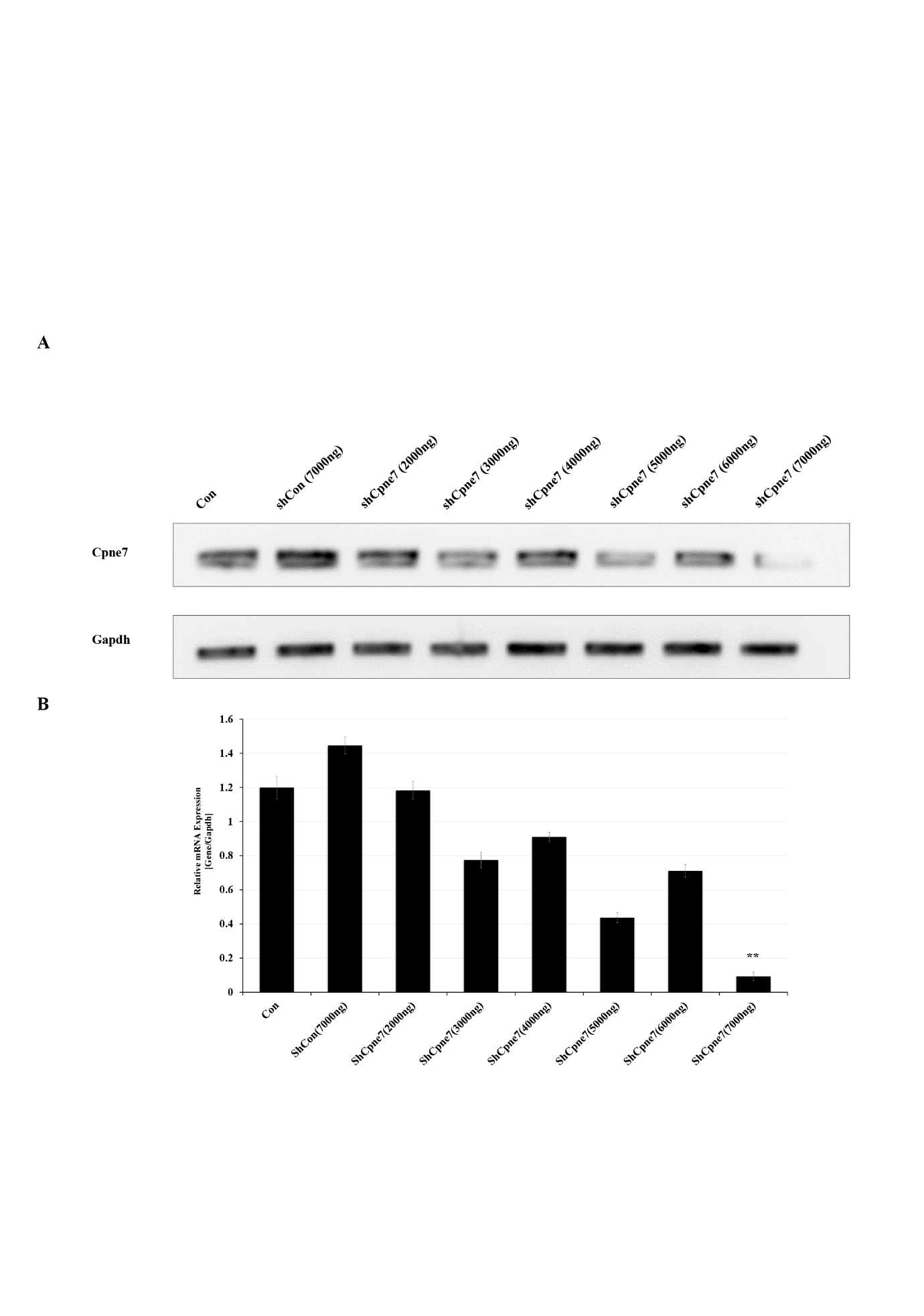


**Figure S7. Downregulation of Cpne7 using the shCpne7 vector in hDPCs.** **(A-B)** Human DPCs were transfected with shCon or shCpne7 for 48h. Cpne7 mRNA levels and semi-quantification were evaluated by real-time PCR. All values represented the mean ± SD of triplicate experiments. **P<0.01 vs Control.


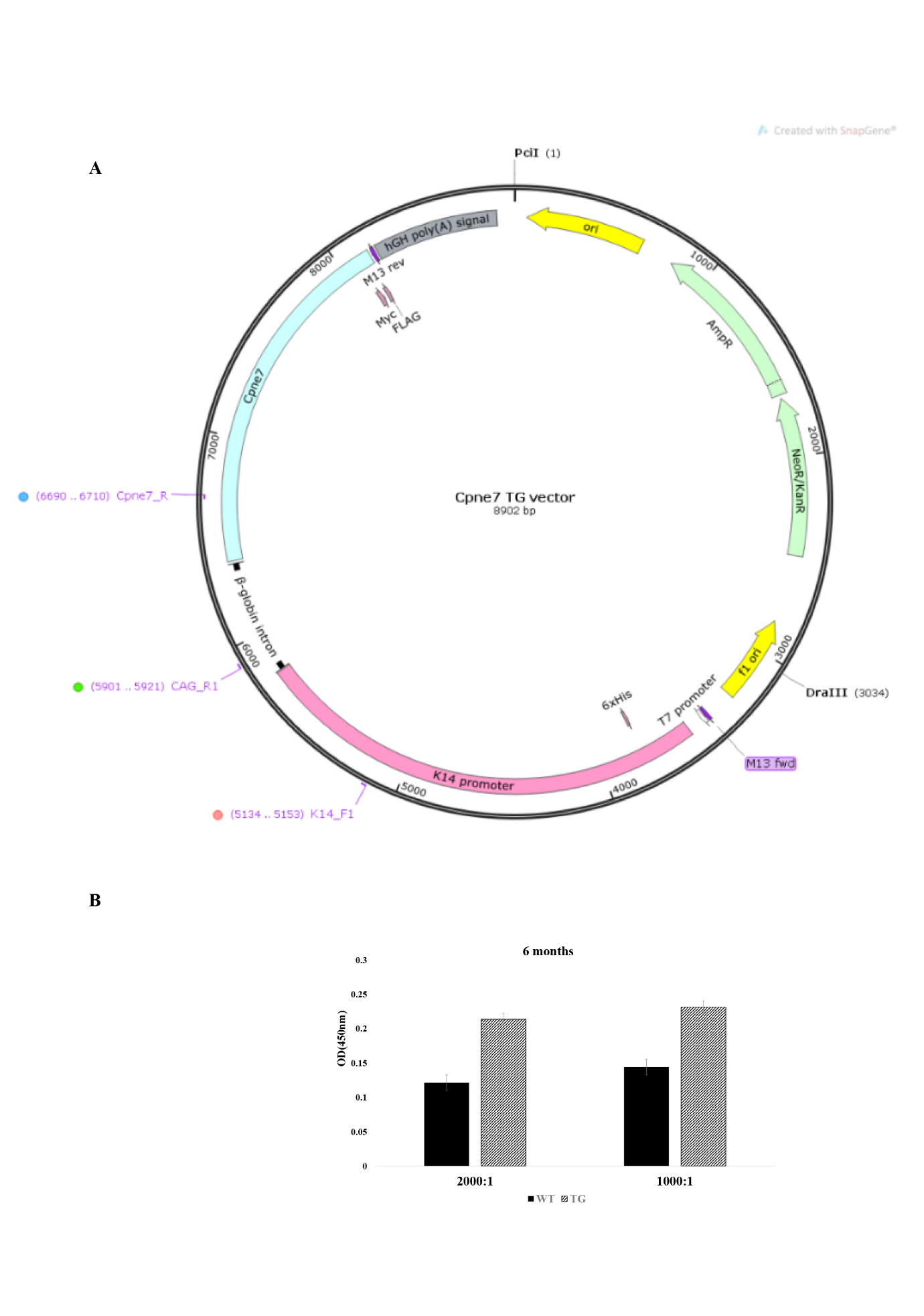


**Figure S8. Generation of Cpne7 transgenic mice by targeted overexpression of Cpne7 gene using K14 promoter. (A)** Construction map of the Cpne7 expression vector with K14 promoter. **(B)** CPNE7 protein expression level was evaluated by ELISA analysis in the whole blood, and semi-quantified. All values represented the mean ± SD of triplicate experiments. *P<0.05 vs Control.


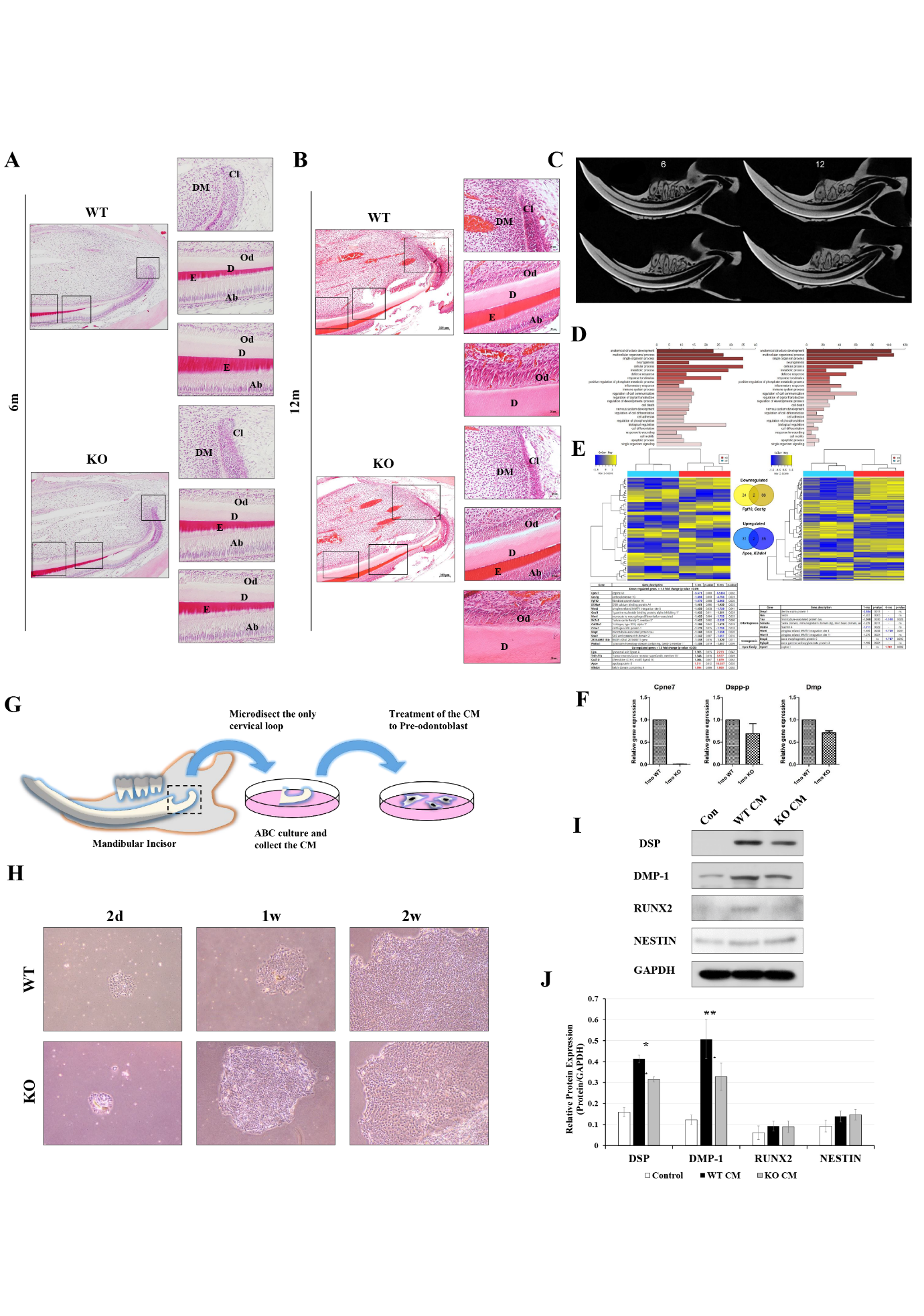


**Figure S9. Cpne7 deletion has no significant effect on incisor development and maintenance. (A-B)** Histological analysis of mandibular incisors of WT and Cpne7^-/-^ mice at 6 and 12 months by H&E staining. Scale bars: 100㎛. Boxed areas were shown at higher magnification. Scale bars: 20㎛. DM, Dental mesenchyme; Cl, Cervical loop; E, Enamel; D, Dentin; Od, Odontoblasts; Ab, Ameloblasts. **(C)** Micro-computed tomographic images of WT and Cpne7^-/-^ mice incisors. **(D)** Bar plot of gene enrichment and functional annotation analysis using gene ontology. **(E)** Heat map construction for hierarchical clustering and a list of downregulated or upregulated genes in Cpne7^-/-^ mice. **(F)** Real-time PCR analysis of odontoblast marker mRNA in the extracted pulp cells of WT and Cpne7^-/-^ mice incisor at 1 month. **(G)** Schematic diagrams of primary culture of apical bud cells (ABCs) from WT and Cpne7^-/-^ mice mandibular incisors, respectively. **(H)** Light microscopic images of ABCs cultured up to 2 weeks from WT and Cpne7^-/-^ mice incisors. **(I-J)** Condition medium (CM) was obtained from cultured WT or Cpne7^-/-^ ABCs. The obtained WT or Cpne7^-/-^ CM was treated into mouse dental papilla cells (MDPC-23 cell line) and cultured for 48 hours. DSP, DMP-1, RUNX2, and NESTIN protein levels were evaluated by western blot analysis, and semi-quantification was obtained. All values represented the mean ± SD of triplicate experiments. *P<0.05 and **P<0.01 vs Control.


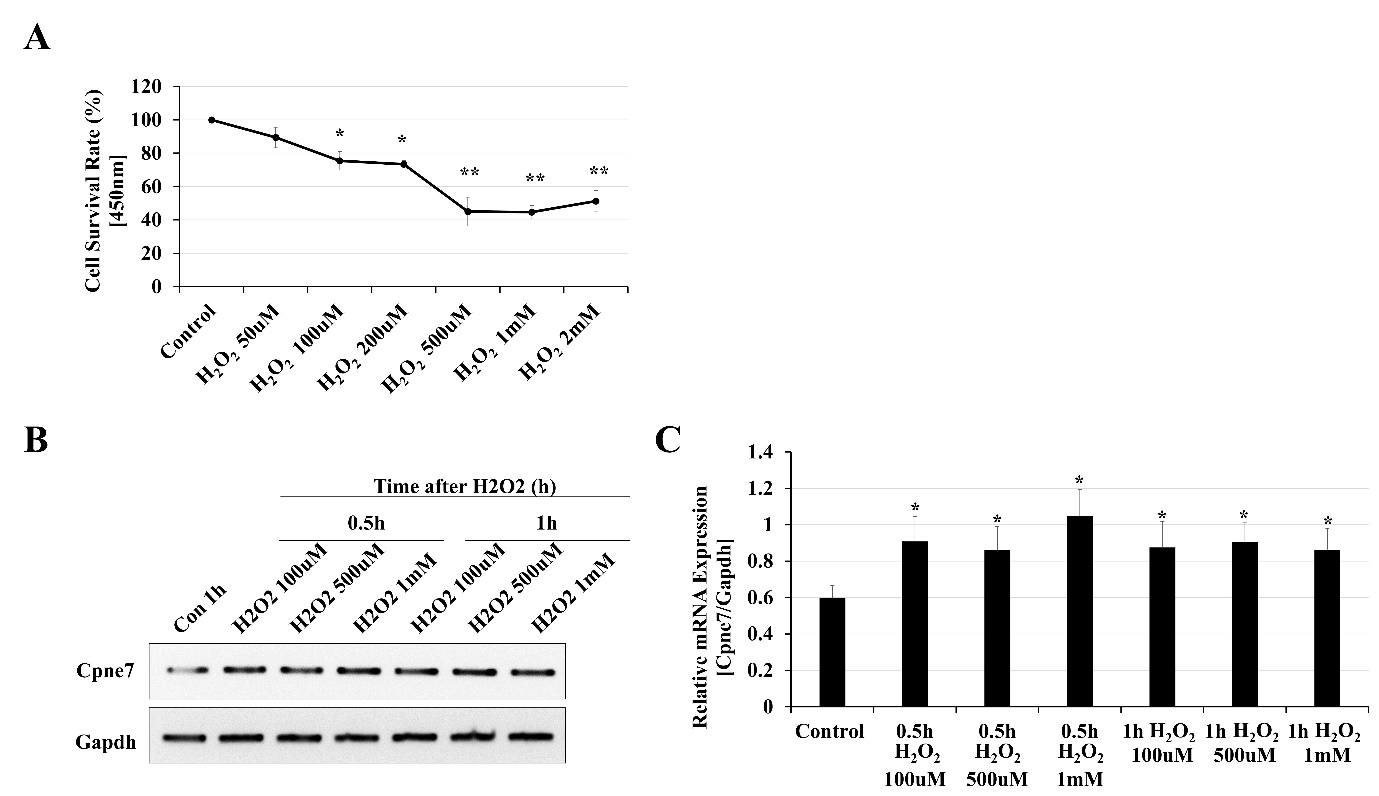


**Figure S10. (A)** Cell survival rate was evaluated by WST-8 analysis in various concentrations of H_2_O_2_-treated hDPCs. **(B-C)** mRNA level of *Cpne7* were analyzed by RT-PCR at 30 minutes or 1h after H_2_O_2_100uM, 500uM, or 1mM treatment in hDPCs, respectively, and semi-quantified. All values represented the mean ± SD of triplicate experiments. *P<0.05 and **P<0.01 vs Control.


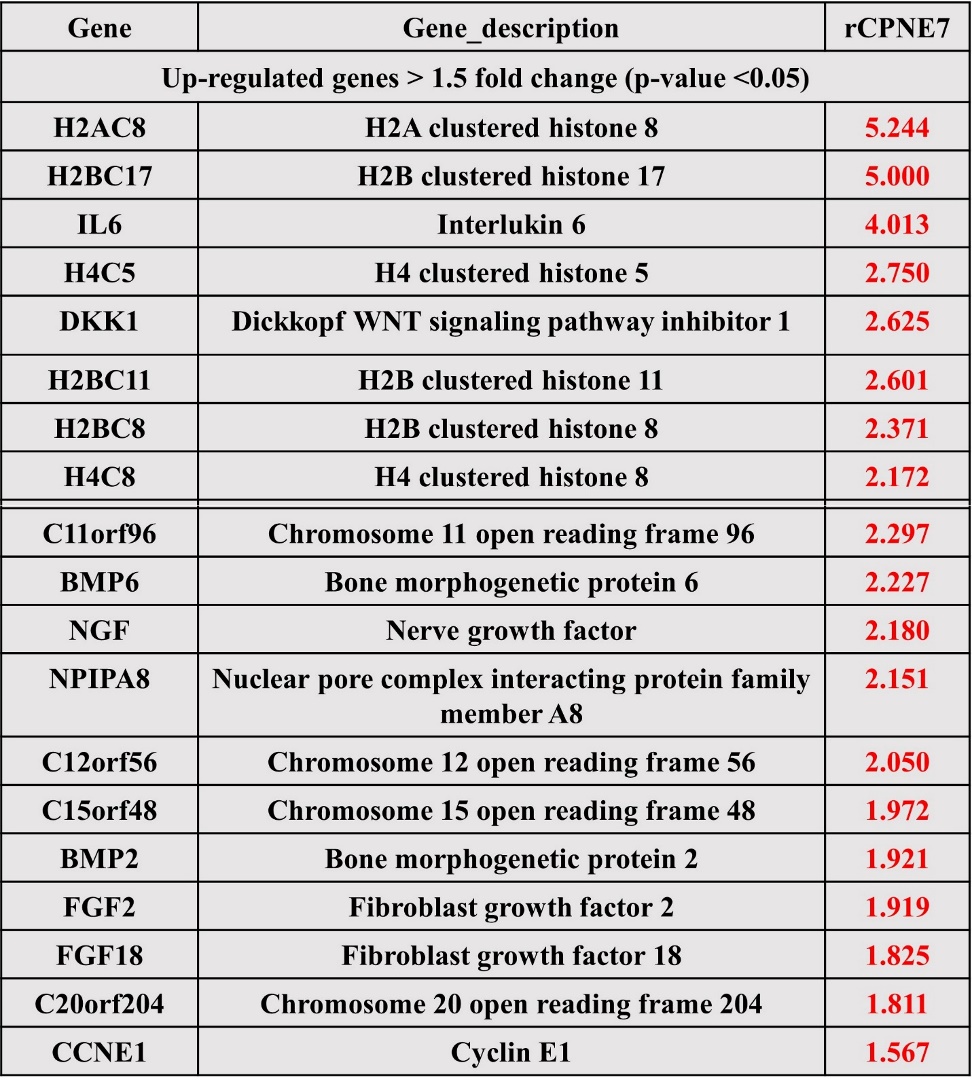


**Figure S11. List of upregulated genes in rCPNE7-treated hDPCs analyzed by RNA sequencing.**


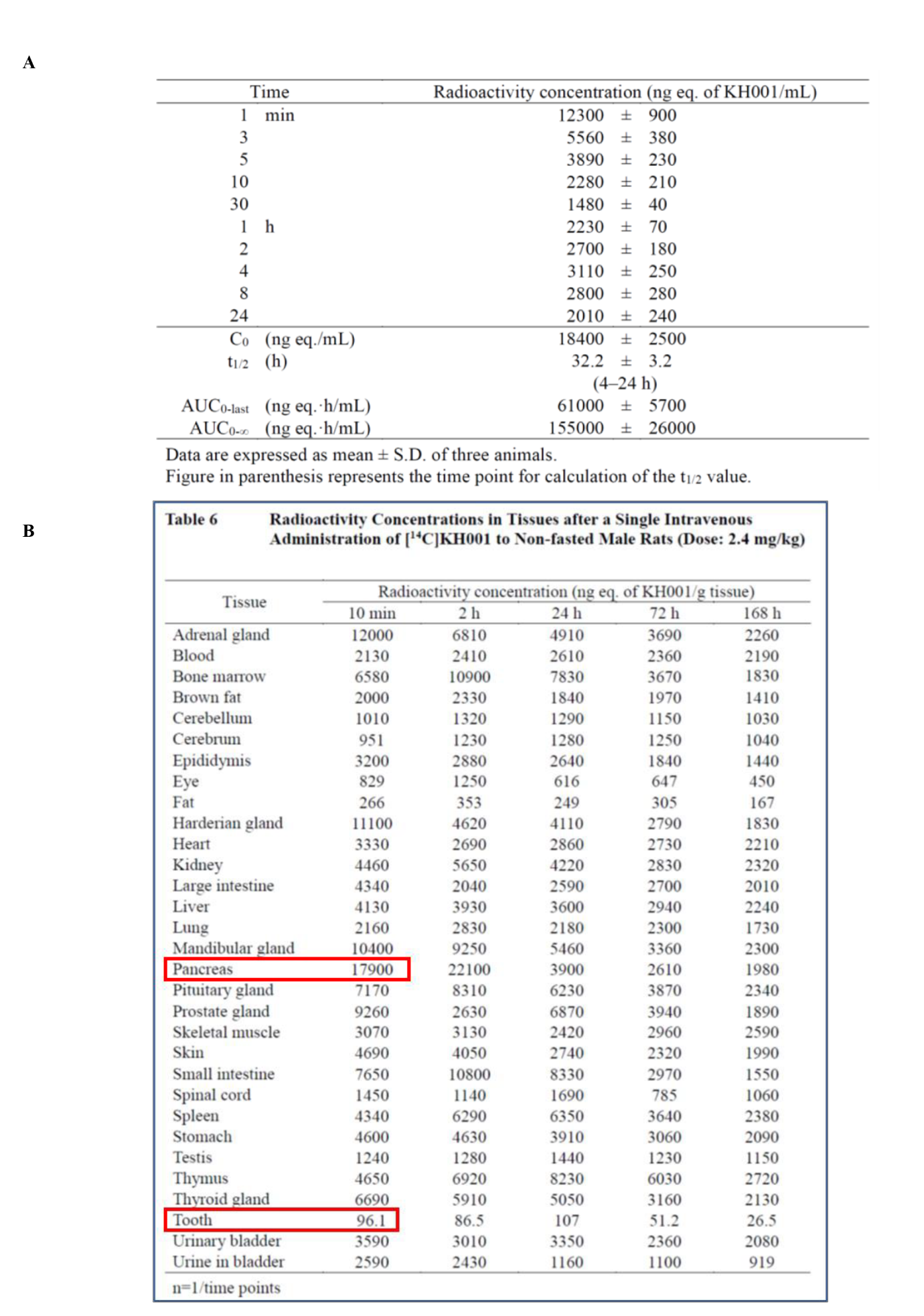


**Figure S12. [^14^C] CPNE7-DP intravenous administration analysis. (A)** Radioactivity in the plasma after a single intravenous administration of [^14^C] CPNE7-DP into non-fasted male rats at a dose of 2.4mg/kg is shown. Initial radioactivity of 18400 ng eq./mL (C_0_), rapidly decreased to 1480 ng eq./mL 30 min after the administration. **(B)** Quantification of the whole body autoradiography. Whole body autoradiograms after a single intravenous administration of [^14^C] CPNE7-DP into non-fasted male rats at a dose of 2.4mg/kg are shown. The radioactivity of the tooth was detected 10 min after the administration.


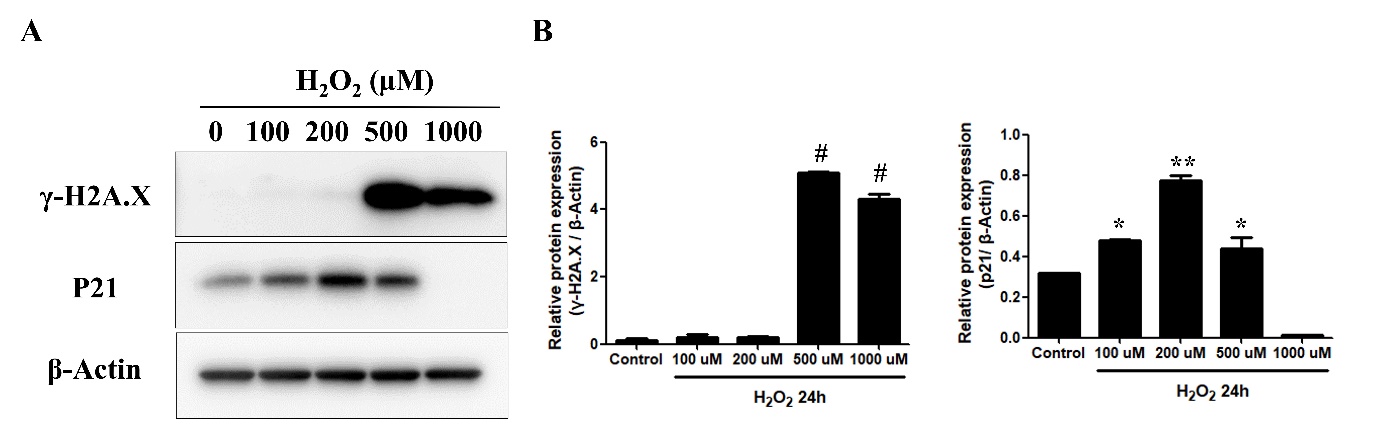


**Figure S13. (A-B)** Gamma-H2AX, and P21 protein levels were analyzed by western blot analysis and semi-quantified in various concentration of H_2_O_2_-treated hDPCs. All values represented the mean ± SD of triplicate experiments. *P<0.05, **P<0.01, and #P<0.0001 vs Control.
